# Segmentation for Learning Adsorption Patterns and Residence-Time Kinetics on Amorphous Surfaces

**DOI:** 10.1021/acs.jcim.5c01463

**Published:** 2025-10-03

**Authors:** Mattia Turchi, Ivan Lunati

**Affiliations:** Laboratory for Computational Engineering, 111825 Swiss Federal Laboratories for Materials Science and Technology, Empa, 8600 Dübendorf, Switzerland

## Abstract

Heterogeneous surfaces such as amorphous silica are characterized by highly heterogeneous local atomic environments that govern the adsorption of gas molecules through spatial arrangements. These surfaces exhibit properties that are particularly relevant for adsorption and catalytic applications. Here, we investigate CO_2_ adsorption landscapes, captured by CO_2_ density maps, which display complex patterns requiring machine learning (ML) segmentation for systematic analysis. We present an optimized segmentation protocol based on a modified Random Forest (RF) classifier designed to control the morphology and spatial extent of the segmented regions via feature smoothing and standardized training parameters. While broadly applicable for specific modeling goals and properties of interest, here, the method is tailored to identify high-density regions that dominate heterogeneous adsorption dynamics. For these regions, we extract residence-time statistics that deviate from exponential behavior, revealing multiple time scales associated with distinct surface defects on amorphous surfaces. The extracted kinetics provide essential information for coarse-grained models of adsorption on disordered surfaces. Such models, parametrized using atomistic simulations, enable the prediction of macroscopically measurable adsorption and desorption rates, which can be directly compared with experiments also under conditions not limited by mass transfer.

## Graphical abstract

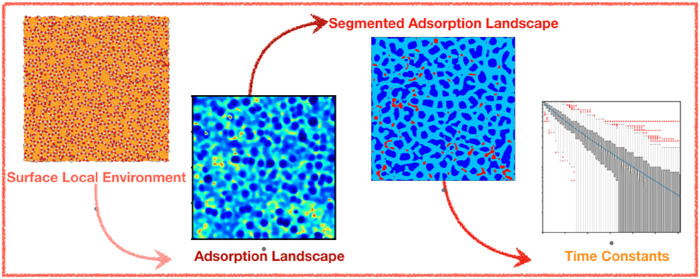

## Introduction

Amorphous materials are widely used in technology and energy applications. For instance, amorphous carbon is used as a hard mask in the semiconductor industry,^1–3^ while amorphous silicates serve as sustainable and nontoxic material for thermal and acoustic insulation.^4–8^ Amorphous oxides and silicates also play an important role in catalysis, both as active materials and as catalytic supports. Amorphous silica, in particular, is widely used as a sorbent and catalytic support,^9–14^ where dispersed undercoordinated defects on the surface can act as preferential anchor sites for metals.^15^ Its broad pore size distribution, spanning micro- and meso-porosity, enhances the accessibility of adsorption and catalytic sites compared to its crystalline counterparts, such as zeolites, which are predominantly microporous.^16^

The reactivity of amorphous silica is strongly influenced by coordination defects, such as undercoordinated silicon (Si3 or E′ centers) and nonbridging oxygen (NBO), which can be introduced by appropriate synthesis protocols.^16–18^ Stable Si3s and NBOs were detected by spectroscopic techniques (i.e., electron paramagnetic resonance, (EPR))^19,20^ and confirmed by ab initio^21–23^ and classical molecular dynamics (MD) simulations.^24^ Their influence on adsorption and catalytic activity has been investigated experimentally^17,25^ and by density functional theory (DFT) simulations.^17,26^

Despite their advantages, amorphous materials remain numerically underexplored because the statistical variability of local atomic environments is difficult to characterize. We recently showed that surface sites exhibit distinct CO_2_ adsorption capacities^27^ and developed a Random Forest (RF) classifier to segment adsorption density maps into high-density (HD), intermediate-density (ID), and low-density (LD) regions.^28^ The classifier used four feature types (intensity, *I*, the magnitude of the intensity gradient, *G*, and the two eigenvalues of the Hessian matrix, *E* ^1^ and *E* ^2^) and four levels of smoothing per feature, for a total of 16 features used for classification.

In this work, we reduce the number of features to four by selecting, based on feature importance and feature correlation analysis, a single smoothing level per feature. This feature reduction allows us to tune the morphology and size of the segmented regions and to test the hypothesis that high smoothing levels for derivative-based features (*G*, *E* ^1^, *E* ^2^) suppress noise and reduce classification uncertainty.

Segmentation of surfaces into discrete classes is essential for developing heterogeneous mesoscale models, such as kinetic Monte Carlo (KMC), which capture the complex dynamics arising from surface site heterogeneity across spatiotemporal scales relevant to adsorption and catalysis.^29–31^

On crystalline surfaces, adsorption density maps are periodic, and desorption typically follows an exponential decay with a single characteristic time, τ, allowing KMC implementations with constant, time-independent transition rates.^29^ In contrast, amorphous surfaces generate highly heterogeneous adsorption landscapes with residence-time distributions significantly deviating from exponential behavior due to spatially confined, highly adsorptive regions.^27,28^ These deviations reflect time-dependent transition probabilities and memory effects, complicating the relationship between residence times and transition rates. Accurately capturing such behavior requires KMC models that explicitly incorporate transitions between subregions (in particular, between HD regions and the surrounding adsorption layer), making reliable surface segmentation a critical prerequisite for kinetic modeling of transport and reaction on disordered surfaces.

In this work, we aim to (i) refine the segmentation procedure to better control the morphology and spatial extent of segmented patterns through careful selection of training data and smoothing levels, (ii) identify strategies that maximize the area of homogeneous classes (i.e., ID and LD), from which desorption follows exponential kinetics; (iii) compute class-specific residence-time statistics for the selected segmentation. As we focus on segmentation, we limit our analysis to residence-time kinetics and consider only adsorption density maps generated by nonreactive MD simulations. Well-defined residence-time constants can be used to infer time-dependent transition rates and parametrize mesoscale kinetic models that enable simulations over larger domains and longer times at tractable computational cost.^32–34^

## Methods

### Segmentation of the CO_2_ Density Maps

We analyze 24 CO_2_ density maps from 100 ns MD simulations of 100 CO_2_ molecules confined in 12 slit pores (2 nm wide), which were reported in our previous work.^28^ Details of the protocol for generating and validating the amorphous surfaces (Table S1 in the Supporting Information), as well as for simulations of CO_2_ adsorption at the pore surfaces, are provided in the Supporting Information. Density maps were generated for both surfaces of each pore. Figure 1 sketches the adsorption of CO_2_ on amorphous silica surfaces and illustrates how different surface sites contribute to the heterogeneity of the adsorption patterns.

**Figure 1:**
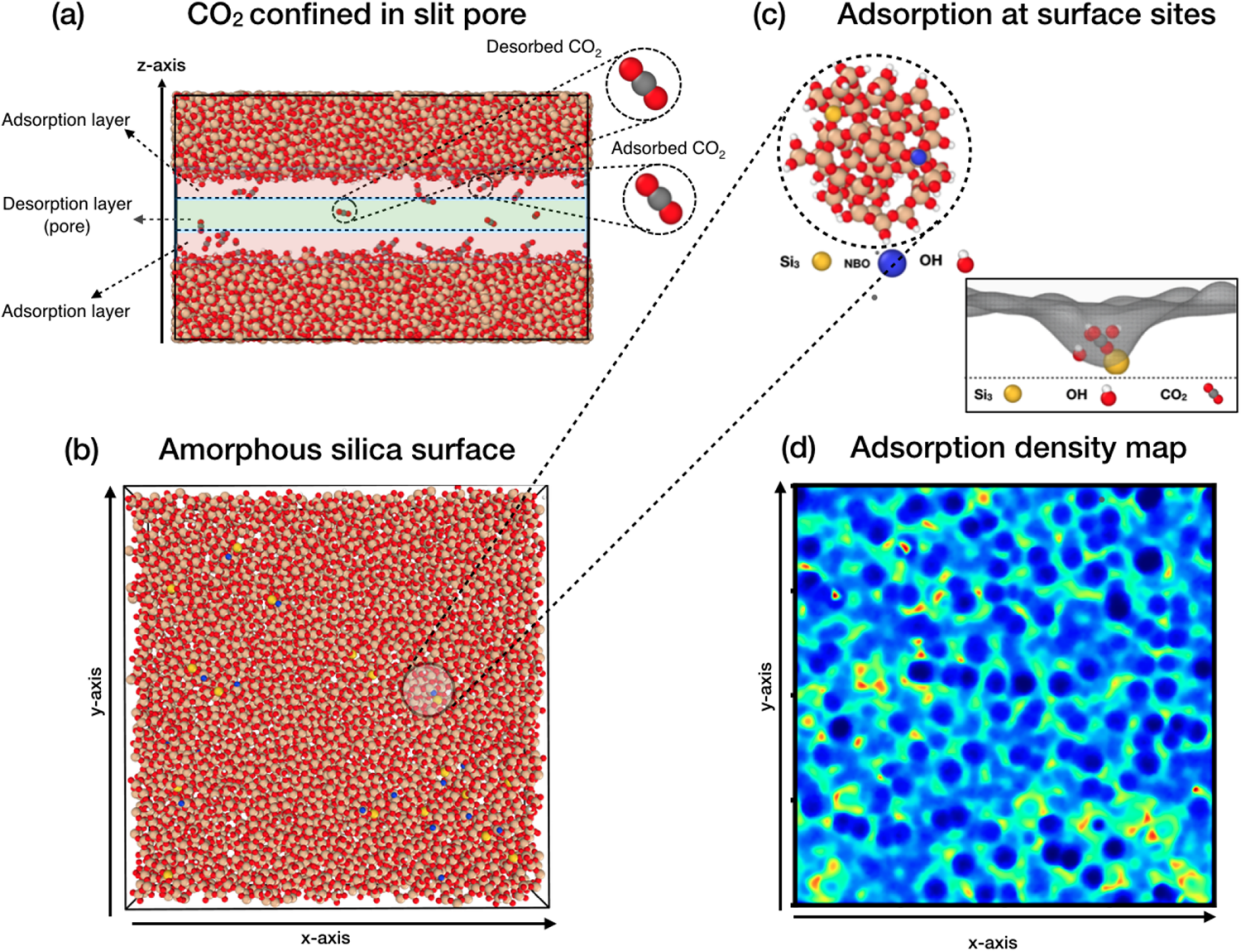
(a) MD snapshot of CO_2_ molecules confined within the 2 nm silica slit pore. Red-shaded regions indicate the adsorption layers, while the green shade identifies the region in the center of the pore where molecules are desorbed and move freely. Adsorption layers are defined from CO_2_ density profiles along the *z*-axis.^28^ Molecules are considered adsorbed as long as they remain in the adsorption layers. (b) Snapshot of an amorphous silica surface (*x*–*y* plane) illustrating atomic speciation and undercoordination defects: oxygen in red, silicon in gold, hydrogen in white, and undercoordinated silicon (Si3) and oxygen (NBO) in yellow and blue, respectively. (c) Local surface environment containing representative adsorption sites (OH groups, Si3, and NBO) and illustration of the adsorption of a CO_2_ molecule near a Si3 site and three OH groups. (d) CO_2_ adsorption density map generated by tracking carbon-atom positions within the adsorption layer, mapped onto a 500 × 500 bin 2D histogram over 90,000 frames from the last 90 ns of the simulation. High-density regions (red/yellow) correspond to undercoordination defects, the intermediate-density region (green/light-blue) forms the adsorption network, and low-density regions (blue/dark-blue) indicate negligible adsorption.

The preprocessing of the density maps (including logarithmic transformation and denoising) and the selection of RF hyperparameters (number of trees and number of drawn features at each split) follows the protocol of ref 28. Density maps are generated by tracking the position of CO_2_ carbon atoms within the adsorption layer, as defined in ref 28. The positions are mapped onto a 2D histogram of 500 × 500 bins over 90,000 frames from the last 90 ns of simulations. For each of the 24 surfaces, the density map is then obtained by averaging the distribution of the adsorbed molecules over time.

We employ the RF classifier, as implemented in the scikit-learn library,^35^ to segment the density maps into three classes of adsorptive regions: low density (LD), intermediate density (ID), and high density (HD). For a supervised algorithm such as RF, the choice of the training data is crucial. In our case, the training set consists of pixels that are chosen from subdomains that have been labeled as belonging to a certain class based on the values of appropriately chosen features. We consider four types of features for each pixel: the intensity (*I*), the magnitude of its gradient (*G*), and the two eigenvalues of its Hessian matrix, *E* ^1^, *E* ^2^. Each feature is smoothed by applying a Gaussian smoothing kernel characterized by the standard deviation (σ). A fixed level of smoothing is applied for *I*(σ = 1) and *G*(σ = 4), while four levels (σ = 1, 2, 3, 4) are tested for *E* ^1^ and *E* ^2^. The choice of testing higher levels of smoothing for first- and second-order derivatives is motivated by the fact that these quantities are more sensitive to noise.^36,37^

Based on the values of *I*, *G*, *E* ^1^, and *E* ^2^, we define three distinct subdomains, *D*_*j*_ with *j* ∈ {LD,ID,HD}, from which the pixels for the training set are sampled and labeled according to the corresponding adsorption class. The sampling subdomains are defined by appropriate cutoff values applied to *E* ^1^, *E* ^2^, *G*, and *I*. The cutoff values of *E* ^1^ and *E* ^2^ are set based on the feature maps of the most smoothed level (σ = 4), Figure 2. The training domains for the three classes are defined as follows:

- LD as the regions close to local minima: small intensity, *I*(σ) < *k*_*I*,LD_, small gradient, *G*(σ) < *k_G_*, and two positive eigenvalues, *E* ^2^(σ) > *k*_*E* ^2^,LD_, *E* ^1^(σ) > *k*_*E* ^1^,LD_;
- ID as the regions close to saddle points: *G*(σ) < *k_G_*, *I*(σ) < *k*_*I*,ID_, and the eigenvalues with different signs, i.e., *E* ^2^(σ) < *k*_*E* ^2^,ID_, *E* ^1^(σ) > *k*_*E* ^1^,ID_;
- HD as the regions close to the local maxima: large intensity, *I*(σ) > *k*_*I*,HD_, small gradient, *G*(σ) < *k_G_*, and two negative eigenvalues, *E* ^2^(σ) < *k*_*E* ^2^,HD_, *E* ^1^(σ) < *k*_*E* ^1^,HD_.

**Figure 2:**
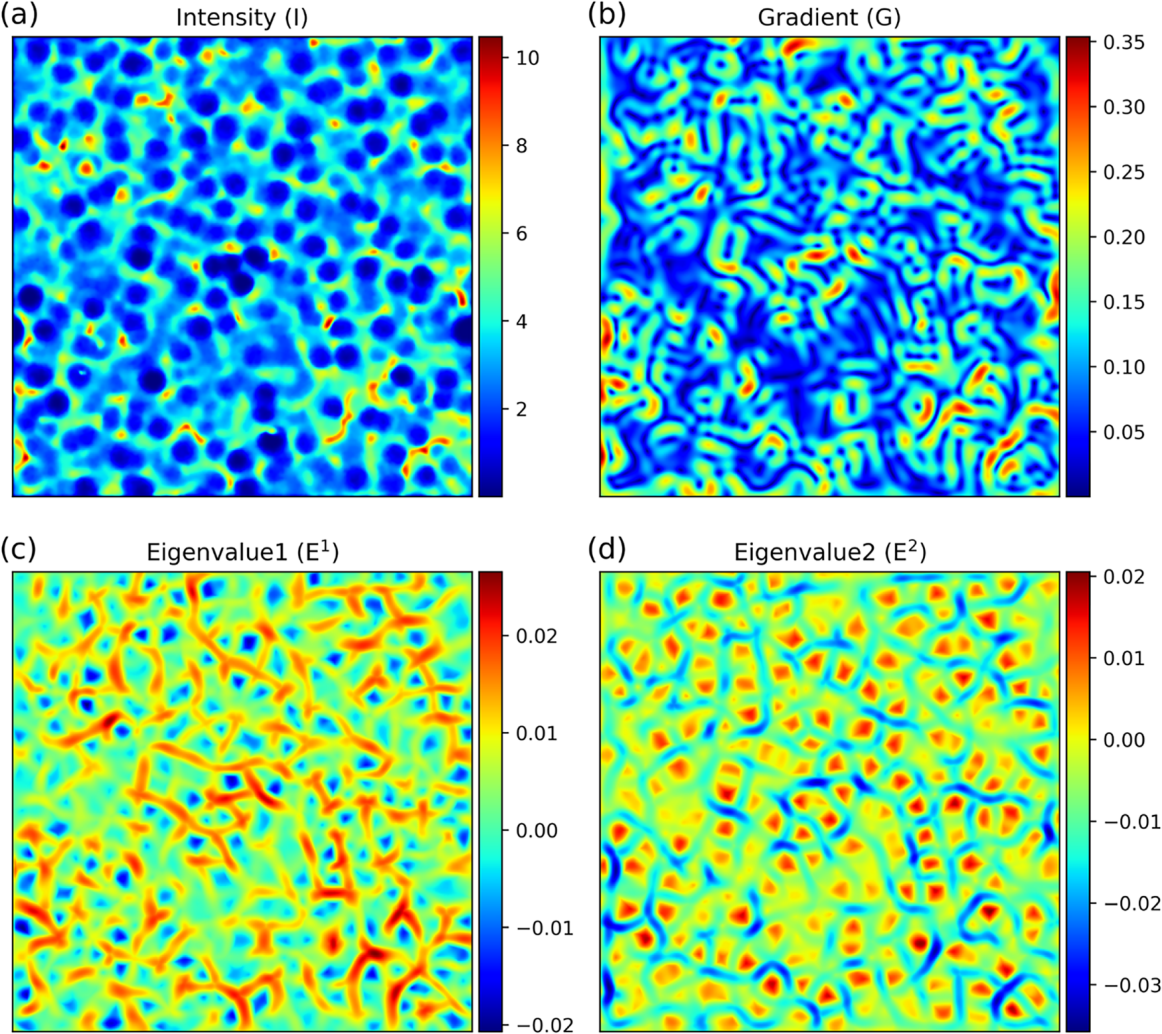
Feature maps used to select the boundaries in the training domains: (a) intensity, *I*(σ = 1), (b) magnitude of the gradient, G­(σ = 4), (c) first eigenvalue, *E* ^1^(σ = 4), and (d) second eigenvalue *E* ^2^(σ = 4).

Compared to our previous work,^28^ we perform a feature importance and correlation analysis to reduce the dimensionality of the classification problem, using the Gini coefficient for importance and the Spearman coefficient for correlation analysis, respectively. Starting from a 16-feature segmentation (comprising *I*, *G*, *E* ^1^, and *E* ^2^, each considered with four different smoothing levels), we assessed the contribution of each feature and their cross-correlation (see Figures S1 and S2 in Supporting Information). This analysis shows that (i) the importance of *I* decreases with increasing smoothing although it remains significant across all levels, (ii) *G* consistently exhibits low importance at all smoothing levels, and (iii) for *E* ^1^ and *E* ^2^, the importance increases with smoothing and becomes significant only at the highest level (σ = 4), which is consistent with the expected noise-reduction effect of smoothing on features computed from higher derivatives. Note that intensity is the only feature type that remains important for all smoothing levels. The correlation analysis further revealed that the four intensities (*I*(σ) with σ = 1, 2, 3, 4) are highly correlated, indicating that considering all of them introduces redundant information and potentially inflates their weight in the classification, consequently weakening the relative importance of the other features.

Based on these observations, we reduce the feature set to four features by selecting a single level of smoothing for each feature type (*I*, *G*, *E* ^1^, and *E* ^2^). This choice minimizes the cross-correlation noise while preserving the most important information. In particular, using a combination containing *E* ^1^(σ = 4) and *E* ^2^(σ = 4) is preferable because only these smoothing levels guarantee the significant importance of these features. As illustrated in Figures S1 and S2, the 4-feature segmentation using *I*(σ = 1), *E* ^1^(σ = 4), and *E* ^2^(σ = 4) significantly increases the relative importance of *E* ^1^ and *E* ^2^ at the expense of *I* with respect to the 16-feature segmentation, resulting in noticeably different segmentation patterns for the LD and ID classes. As demonstrated in the result section, this configuration also leads to a reduced pixel-classification uncertainty, quantified based on the Shannon entropy metric (*H*), and provides better control over the morphology and spatial extent of the segmented regions as a function of the applied level of smoothing.

We consider two groups of RF segmentations, characterized by two different cutoffs applied to the intensity feature (*I*) for the ID class, i.e., *k*_*I*,ID_ = 3 and *k*_*I*,ID_ = 5. In the following, these two segmentation groups are referred to as RF(3) and RF(5), respectively. In contrast to our previous work,^28^ the cutoffs applied to all features are kept fixed and only the smoothing levels are varied across different segmentations. This choice enhances the robustness of the segmentation algorithm, yielding segmentation patterns that consistently and systematically depend only on the smoothing level. To benchmark the performance of the RF classifier, we also considered a third segmentation group, denoted by THR, based only on simple thresholding in the *E* ^1^ – *E* ^2^ space. For each segmentation group, we fix the smoothing level on the intensity and gradient magnitude to *I*(σ = 1) and *G*(σ = 4), respectively, and assess the effects of smoothing on the two Hessian eigenvalues. Specifically, we consider all four smoothing levels for each eigenvalue, which results in 16 combinations of the four feature types.

### Residence-Time Analysis

Once the density maps have been segmented, we calculate the residence-time distribution in the segmented regions for the molecules adsorbed in the surface layer (defined as in ref 28). Following the approach in ref 28, we consider multiple initial time frames and track adsorbed molecules over successive time windows of 400 ps, hence, over successive 400 frames separated by 1 ps. To improve the statistics of the adsorption events, a total of 225 nonoverlapping time windows are considered from the final 90 ns of the simulation.

In calculating the residence-time distribution, we follow a procedure different from that in our previous work. Rather than measuring the total time that each molecule spends in the ID class, we merge the ID and LD regions, defining the background-density class BD = ID ∪ LD, and calculate the residence time within the BD domain before either undergoing a transition to the HD class (BD → HD) or being desorbed (BD → pore). The ID and LD classes are merged to avoid fragmentation of the residence time, which may result from molecules occasionally traversing the LD regions. Although the residence time in LD is typically short, such crossings are not uncommon due to the large number and spatial extension of LD regions.

For molecules adsorbed in HD regions, we compute the total residence time within each individual HD region before the transition to the BD class (HD → BD) or desorption to the pore (HD → pore) occurs. Given the limited size of individual HD regions, this assumption is reasonable. Occasional short-term exits and re-entries into the same HD region (after a few frames) are caused by a few pixels at the region boundary being classified as ID and are not to be considered true desorption events. This approximation is justified by the fact that density maps are averaged over 90,000 frames, with about 40 CO_2_ molecules adsorbed per frame on average. Therefore, a definition of the boundaries among the different classes that suits the individual CO_2_ trajectories is practically not feasible.

The residence-time distribution of each molecule within the BD and HD regions is fitted using a biexponential function

1 $$\frac{N\left(0\right)-N\left(t\right)}{N\left(0\right)}=a\exp\left(-\frac{t}{\tau_{1}}\right)+\left(1-a\right)\exp\left(-\frac{t}{\tau_{2}}\right)$$

where *N*(*t*) is the number of molecules with total residence times larger than *t* and *N*(0) is the number of molecules adsorbed at time *t* = 0. In eq 1, the parameter *a*, respectively (1 – *a*), is the fraction of molecules characterized by a time constant τ_1_, respectively τ_2_. The parameter *a* informs about how much the residence-time distribution is close to an exponential behavior. The relative mean adsorption time (or mean lifetime of the molecule within the region of interest) is

2 $$\mathrm{\tau ̅}=a\tau_{1}+\left(1-a\right)\tau_{2}$$

The uncertainty in the fitting parameters is estimated as the standard deviation obtained from the diagonal elements of the covariance matrix, and the error in τ̅ is computed using standard error propagation rules.

We note that, unlike the case of an exponential residence-time distribution, a biexponential distribution implies a time-dependent transition probability. While this renders the relationship between residence-time constants and transition rates nontrivial, it highlights the value of segmentation: isolating regions that deviate from exponential behavior enables a targeted investigation of heterogeneous adsorption dynamics, distinct from background regions governed by constant-rate and memoryless processes.

## Results

### Spatial Extent and Morphology of the Segmented Regions

We begin by comparing the spatial extent and morphological features of the ID, LD, and HD regions resulting from three segmentation approaches: RF-based classification with two different intensity cutoffs on the intensity defining the ID class (*k*_*I*,ID_ = 3 and *k*_*I*,ID_ = 5) and segmentation by direct thresholding in the *E* ^1^ – *E* ^2^ feature space. The remaining cutoff values applied to the four features are summarized in Table 1. For all three approaches in this analysis, the highest level of smoothing (σ = 4) is applied to the two eigenvalues of the Hessian matrix (i.e., *E* ^1^(σ = 4) and *E* ^2^(σ = 4)).

**Table 1:** Cutoff Values Applied to the Features of the Three Segmentation Groups^a^

	RF(3)			RF(5)			THR	
	*k*_*E* ^2^_	*k*_*E* ^1^_	*k_I_*	*k*_*E* ^2^_	*k*_*E* ^1^_	*k_I_*	*k*_*E* ^2^_	*k*_*E* ^1^_
LD	>−0.005	>−0.005	<1.0	>−0.005	>−0.005	<1.0	>−0.005	>−0.005
ID	<−0.003	>−0.005	<3.0	<−0.003	>−0.005	<5.0	<−0.005	>−0.005
HD	<−0.003	<−0.004	>7.0	<−0.003	<−0.004	>7.0	<−0.005	<−0.005

a (i) RF with cutoff to intensity for ID class at 3 (RF(3)) and 5 (RF(5)) and threshold segmentation (THR).

Figure 3 shows the distribution of pixels attributed to the three classes (LD, ID, and HD) in the *E* ^1^(σ = 4) – *E* ^2^(σ = 4) feature space, as well as the corresponding segmented density maps for a reference surface (the same surface as in Figure 2).

**Figure 3:**
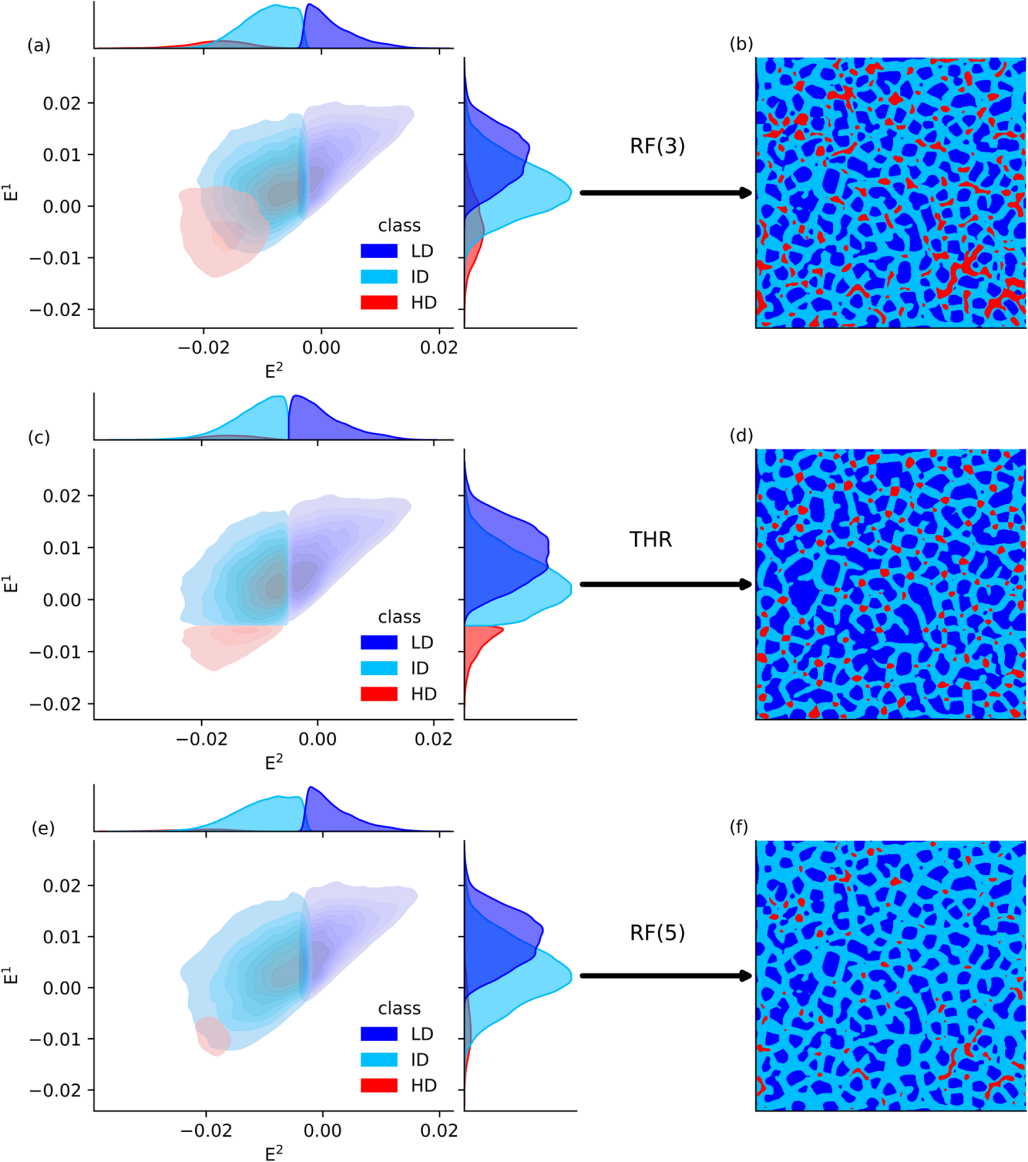
Sampling domain in the *E* ^1^(σ = 4) – *E* ^2^(σ = 4) space and the resulting segmented density map for the three segmentation groups: RF(3) (panels a, b), *THR* (panels c, d), and RF(5) (panels e, f).

We notice that three segmented maps display notably different distributions of the three classes, particularly in the number and extent of HD regions. With a lower intensity cutoff for the ID class, *k*_*I*,ID_ = 3, a large portion of the density map is assigned to the HD class. Raising the cutoff value to *k*_*I*,ID_ = 5 drastically reduces the extent of extension of the HD regions in favor of the ID class, while the LD regions remain similar in the two cases. Both RF segmentations capture the irregular nonconvex morphology of the HD regions well (Figure 2a). In contrast, THR segmentation yields an intermediate extension of the HD regions (between the extension of RF(3) and RF(5)) but fails to identify elongated HD patterns. This limitation originates from the sharp nonoverlapping thresholds applied to the two eigenvalues (*k*_*E* ^1^_ = *k*_*E* ^2^_ = −0.005) in the *E* ^1^ – *E* ^2^ feature space (Figure 3c) and from the absence of cutoffs on the intensity feature of the three classes. For the LD regions, THR identifies the LD regions that are more extended and compact than in the RF segmentations, again due to the strict nonoverlapping boundaries in the eigenvalue feature space.

### Effects of Feature Smoothing

The spatial extent and morphological characteristics of the LD, ID, and HD regions are significantly affected by the degree of smoothing applied to the eigenvalue-based features (*E* ^1^, *E* ^2^), which determines the scale at which structural heterogeneities are captured. Lower smoothing levels tend to preserve fine-grained fluctuations, resulting in more fragmented and irregular domains, while higher levels suppress noise and favor the emergence of larger, more coherent regions. While we fix the smoothing levels on the intensity and the gradient to σ = 1 and σ = 4, respectively, we compare 16 combinations of the smoothing levels for the two eigenvalues (with σ = 1, 2, 3, 4), both for the RF segmentations and simple thresholding.

Figure 4 presents the statistics of the total surface areas of the three density classes (LD, ID, and HD) obtained for the 24 surfaces segmented using different levels of smoothing. Results are shown for all three segmentation groups (RF(3), RF(5), and THR). As LD regions are defined by positive values of *E* ^2^, the smoothing level applied to the *E* ^2^ feature influences the surface area assigned to the LD class. In all three segmentation groups, the area assigned to the LD class tends to increase with σ and reaches a maximum at *E* ^2^(σ = 4) (Figure 4a–c). In contrast, the total surface area of the LD regions is largely insensitive to the smoothing level applied to *E* ^1^. Higher σ values also result in more compact LD regions (Figure 5).

**Figure 4:**
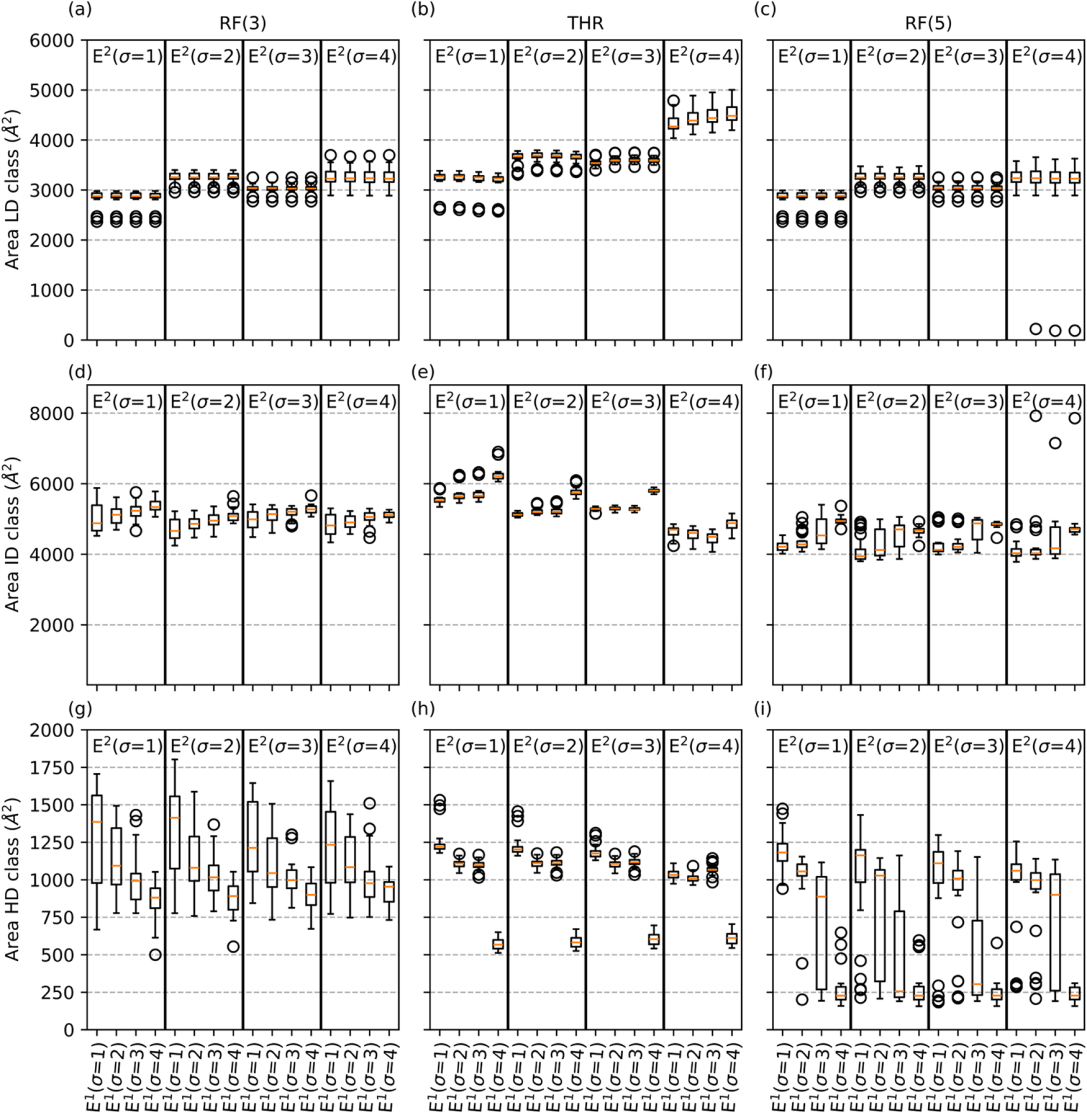
Effect of the smoothing levels of *E* ^1^ and *E* ^2^ on the area attributed to LD, ID, and HD for the three segmentation groups: RF(3) (panels a (LD), d (ID), and g (HD)), THR (panels b (LD), e (ID), and h (HD)) and RF(5) (panels c (LD), f (ID), and i (HD)). The box plots represent the 0.25–0.75 quartiles (with whiskers extending to 1.5 times the inner quartile range); median values are depicted in orange.

**Figure 5:**
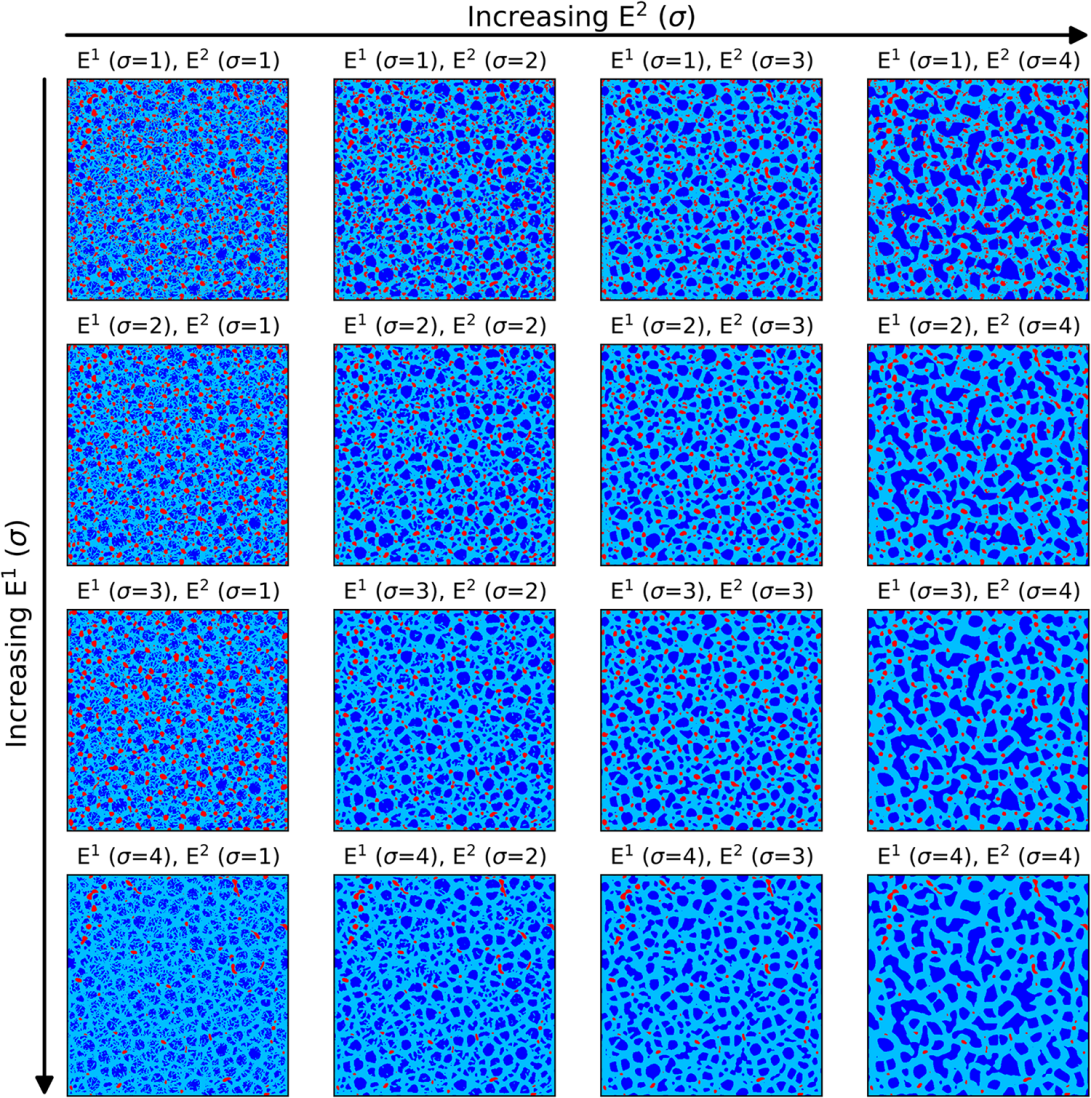
Example of segmentation results for one of the 24 surfaces using RF(5) with the 16 different smoothing levels (σ). From left to right, increasing the smoothing of the second eigenvalue (*E* ^2^(σ)) yields more compact LD regions (blue). From top to down, increasing the smoothing of the first eigenvalues (*E* ^1^(σ)) reduces both the size and the number of HD regions (red) while increasing the extension of the ID class (light blue).

The sign of *E* ^1^ governs the partitioning of the remaining domain between the ID and HD classes, thus influencing the total surface area of these regions (Figure 4), as well as the number of disconnected HD regions on each surface (Figure 5). Increasing the level of smoothing of the *E* ^1^ feature results in a systematic reduction in the total surface area assigned to the HD regions, with a corresponding expansion of the ID regions. This effect is especially pronounced when comparing *E* ^1^(σ = 4) with lower smoothing levels. Segmentations with *E* ^1^(σ = 4) also exhibit a reduced variance of the total surface areas of both ID and HD classes than segmentations with a lower level of smoothing, suggesting that smoothing enhances the consistency of the spatial partitioning across surfaces as a result of noise reduction on the *E* ^1^ and *E* ^2^ features.

#### Uncertainty of RF Segmentation

RF segmentation assigns each pixel to one of the three classes (LD, ID, or HD) by a majority vote across decision trees. For each pixel, the algorithm provides a probability vector *p* = [*p*_LD_, *p*_ID_, *p*_HD_], where *p*_*j*_ = *M*_*j*_/*M* represents the fraction of trees that classifies the pixel in class *j*, *M*_*j*_ being the number of trees that classifies the pixel in class *j*, and *M* being the total number of trees. The pixel is then assigned to the class with the highest *p*_*j*_. To quantify the uncertainty of this classification, we compute the Shannon entropy *H* from the probability vector

3 $$H=-\sum_{j=1}^{3}\left[p_{j}\ln\left(p_{j}\right)\right]$$

where high values of *H* correspond to pixels with highly uncertain classification, and *H* reaches its maximum when the classification is most uncertain (i.e., *p*_*j*_ = 1/3 for all *j*) and is zero for fully confident assignments (i.e., *p*_*j*_ = 1 for one class and zero for the others). Notice that the quantification of classification uncertainty is a clear advantage of using RF over thresholding, for which the Shannon entropy cannot be computed.

To quantify the uncertainty of classification at the class level, we calculate the median values of the Shannon entropy (*H̃*) for all pixels assigned to each class.^38^ Figure 6 displays *H̃* values for RF(3) and RF(5) segmentations. Among the three classes, HD is the most sensitive to the level of smoothing, displaying *H̃* values that vary significantly with *E* ^1^(σ) and range from values close to zero to approximately 0.7. For RF(5), the combination *E* ^1^(σ = 4) and *E* ^2^(σ = 4) yields the lowest *H̃*, with minimal variance and no outliers. In general, all combinations with *E* ^1^(σ = 4) exhibit lower *H̃* values compared to others. For RF(3), the lowest *H̃* is observed with *E* ^1^(σ = 3) and *E* ^2^(σ = 4) although this combination shows a few outliers.

**Figure 6:**
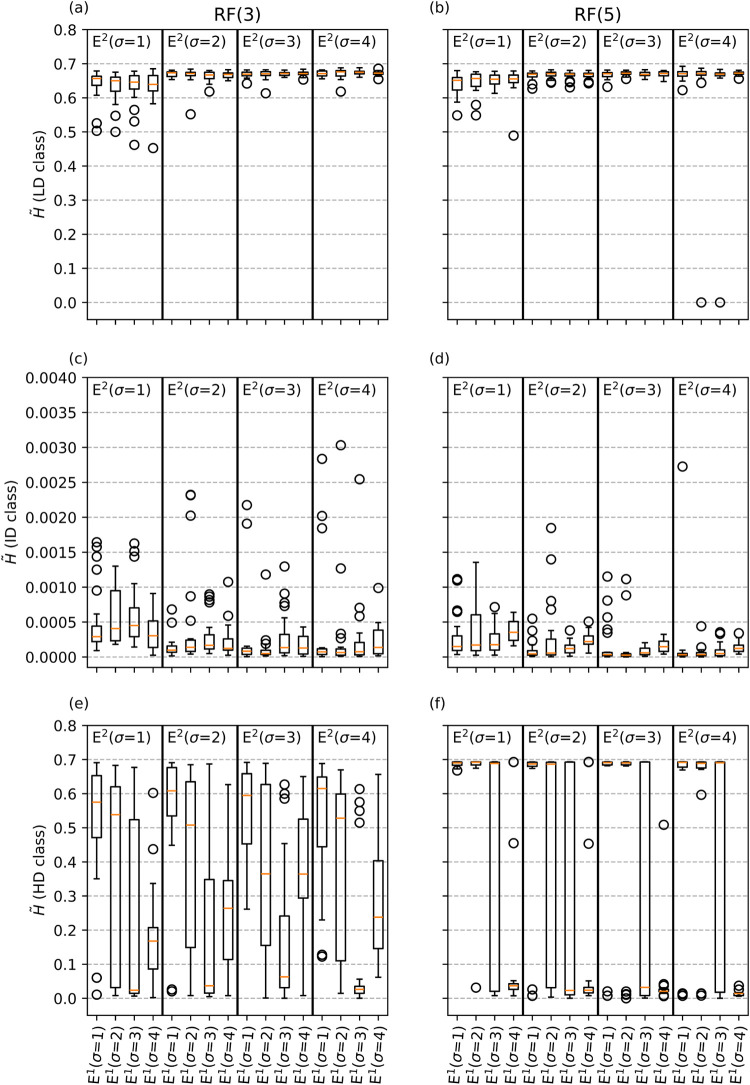
Distribution of the median values of Shannon Entropy (*H̃*) computed over the 24 surfaces for the three segmentation classes (LD, ID, and HD) and the different combinations of *E* ^1^ and *E* ^2^. Analysis is done on the segmentation performed with RF classifiers RF(3) (panels a (LD), c (ID), and e (HD)) and RF(5) (panels b (LD), d (ID), and f (HD)). The box plots represent the 0.25–0.75 quartiles (with whiskers extending to 1.5 times the inner quartile range), and median values are depicted in orange.

When the two RF segmentations are compared with different levels of smoothing, no significant differences are observed for the LD class (Figure 6a,b), for which *H̃* remains relatively high. This persistent uncertainty is attributed to the classification of LD being based only on the *E* ^2^ feature, as well as to the overlap, in terms of training regions, of the *I* feature values with those of the ID class. Indeed, in terms of the *I* feature, all training data for the LD class fall within the region from which also ID training data are sampled: 0 < *k*_*I*,LD_ < 1 and 0 < *I*_ID_ < 3 (respectively 5) for RF(3) (respectively RF(5)). In contrast, the classification uncertainty is consistently low for the ID class across all smoothing levels (Figure 6c,d), which can be explained by the cutoff applied to the *I* feature (the training data labeled as ID with *I* > 1 have no overlap with the sampling region of the LD class). For the HD class, a marked reduction in classification uncertainty is observed when *E* ^1^(σ = 4) is employed (Figure 6e,f), highlighting the importance of sufficient smoothing for this feature.

Compared to our previous work,^28^ we observe a substantial reduction in classification uncertainty for the ID class across all levels of smoothing. Also for the HD class, *H̃* values are markedly and consistently lower. Two factors contribute to this improvement: (i) we trained the RF classifier on fewer features (four, one per feature type), reducing the variability introduced by considering multiple levels of smoothing for each feature in the classification task; (ii) increased levels of smoothing reduce noise on second derivatives that are crucial classification features (*E^1^* and *E* ^2^), accounting for the abrupt reduction of *H̃* observed using *E* ^1^(σ = 4) for HD.

The uncertainty analysis will be used as one of the criteria to select the segmentation configuration to be used to calculate the mean residence time in the BD and HD classes.

### Mean Residence Time and Transition across Segmented Subdomains

#### Transitions from BD to the Pore and to HD

Random Forest (RF) segmentation learns adsorption patterns of complex morphology, which can be employed to construct models that capture adsorption kinetics on amorphous surfaces by describing transitions between heterogeneous subdomains before they desorb to the pore. Here, we calculate the residence-time constant for the HD and BD regions, hence merging the LD and ID classes into the latter. This merging mitigates the fragmentation of the residence time arising from molecules briefly crossing the LD regions during prolonged residence in the ID region. Although individual residence times in LD are short, they occur rather frequently due to the large spatial extent and fragmented morphology of the LD regions. The residence time is computed conditionally on the region to which the molecule ultimately transitions, either the pore or the HD region. If the transition probabilities are time-independent and each molecule can transition to either state at any time, the two distributions should be identical. Any systematic difference between them signals time-dependent rates. To account for this, we fit the residence-time distributions using a biexponential function, eq 1, which captures possible deviations from single-exponential behavior, and calculate the mean residence time (τ̅) from the two residence-time constants (eq 2).

Among all levels of smoothing tested for the three segmentations (THR, RF(3), and RF(5)), we select two combinations of smoothing levels for the eigenvalues, i.e., *E* ^1^(σ = 3) – *E* ^2^(σ = 4) and *E* ^1^(σ = 4) – *E* ^2^(σ = 4). These smoothing parameters were chosen because (i) they correspond to the segmentation with the lowest Shannon entropy *H̃* for the HD class specifically, *E* ^1^(σ = 3) – *E* ^2^(σ = 4) for RF(3) and *E* ^1^(σ = 4) – *E* ^2^(σ = 4) for RF(5) (Figure 6), and (ii) they produce substantially different areas attributed to ID and HD (Figure 4).

Figure 7 displays the distributions of the two residence-time constants (τ_1_ and τ_2_) and the fraction, *a*, of molecules following τ_1_ for the 24 surfaces and three segmentation groups. Figure 7a,b show transitions from BD to the pore for the two smoothing levels, while Figure 7c,d show transitions from BD to HD. The fitting constants and relative errors are reported in Tables S2–S7. We observe that the combination *E* ^1^(σ = 4) – *E* ^2^(σ = 4) produces higher median values and a lower standard deviation for both τ_1_ and *a* in all segmentation groups. Among the three segmentation groups, RF(5) consistently produces the highest values of τ_1_ and *a*. A similar trend is observed for the BD → HD transition (compare Figure 7d with Figure 7c).

**Figure 7:**
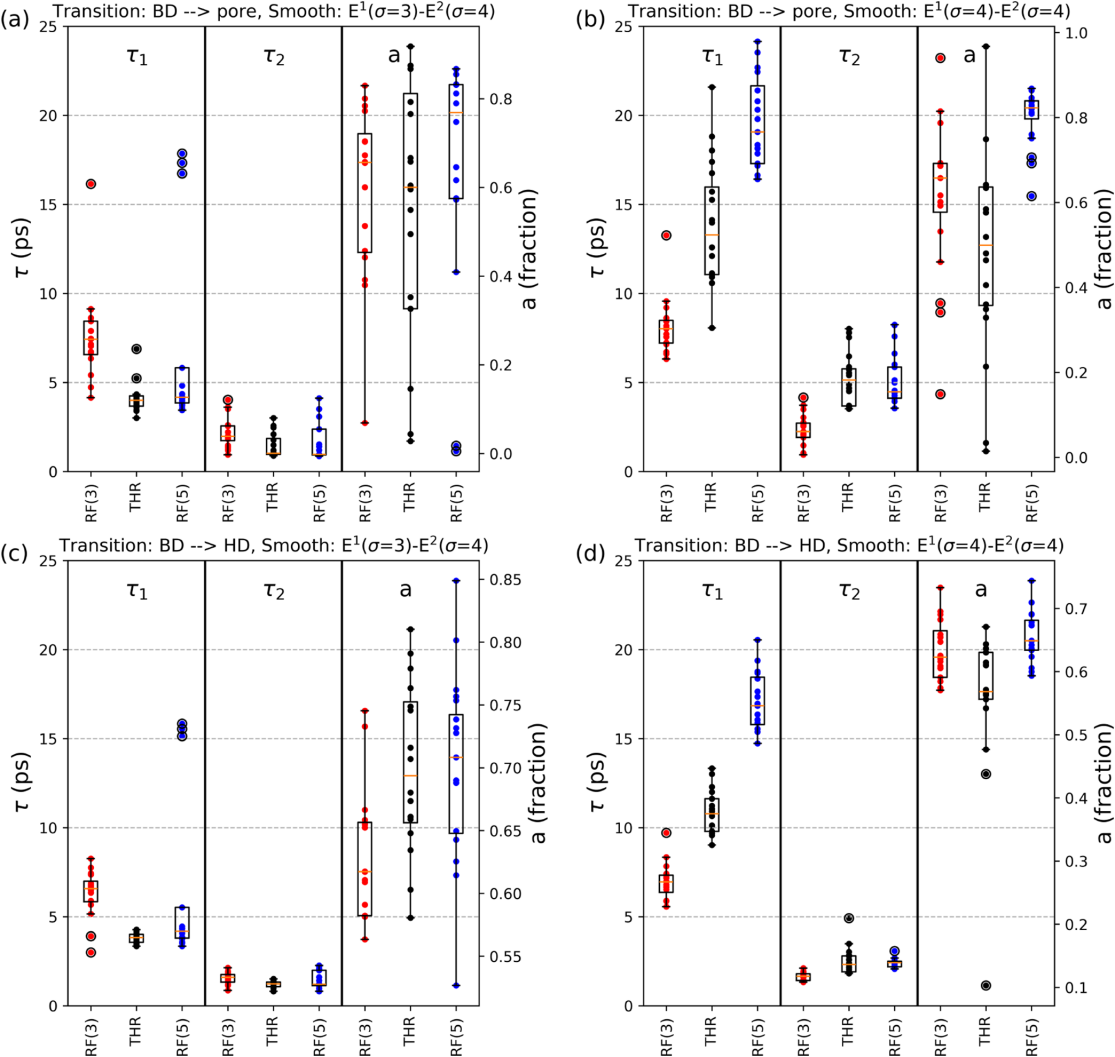
Distributions of the two residence-time constants (τ_1_, τ_2_) and the fraction of molecules following τ_1_ (a) for the 24 surfaces and the three segmentation groups; values are computed by means of eqs 1 and 2. Panels (a, b) are relative to the BD → pore transitions for *E* ^1^(σ = 3) – *E* ^2^(σ = 4) respectively *E* ^1^ (σ = 4) – *E* ^2^(σ = 4); panels (c, d) consider BD → HD transitions for *E* ^1^(σ = 3) – *E* ^2^(σ = 4) respectively *E* ^1^(σ = 4) – *E* ^2^(σ = 4). The box plots represent the 0.25–0.75 quartiles (with whiskers extending to 1.5 times the inner quartile range), and median values are depicted in orange.

In the two transitions (i.e., BD → pore versus BD → HD), the parameter *a* is always higher for the BD → pore transition, indicating that the corresponding residence-time distributions are closer to a single-exponential form. In contrast, transitions from the BD class to the HD regions display a more pronounced deviation from a single-exponential behavior, reflecting the heterogeneity of the HD regions, which are characterized by different adsorption capacities. In general, the combination of *E* ^1^(σ = 4) – *E* ^2^(σ = 4) for the segmentation RF(5) yields higher values of τ_1_ and *a*.

#### Reclassification of HD Regions to Improve Residence-Time Fitting

Using the RF(5) segmentation with the smoothing combination of *E* ^1^(σ = 4) – *E* ^2^(σ = 4), we investigate whether reclassifying part of the HD regions into the BD class could improve the description of the BD → pore transition by a single exponential (Figure 8a,c,e). We recall that this is crucial if we want to isolate regions where transition rates are time-dependent, potentially exhibiting memory effects and necessitating a more careful residence-time analysis for the construction of accurate kinetic models. We recursively reassign to the BD class those HD regions that are visited for less than a certain fraction of time and then recompute the residence-time distribution (fitting parameters for each iteration are reported for a reference surface in Table S8).

**Figure 8:**
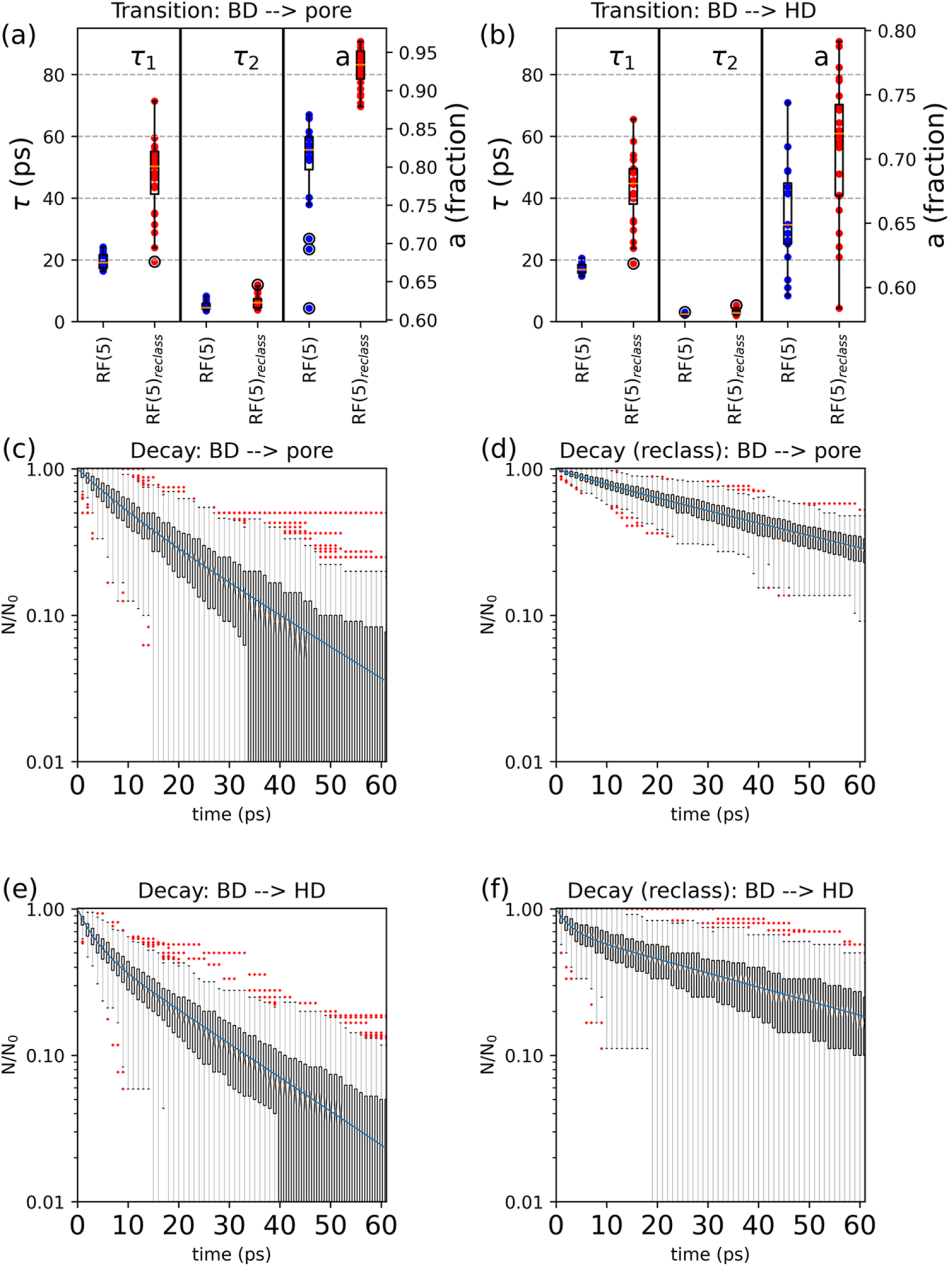
Change in the distribution of decay constants for the 24 surfaces (a, b) and residence-time distributions (for a reference surface) of the BD → pore (c and d) and BD → HD (e, f) transitions when part of the HD regions are reclassified as BD to achieve a closer fit to a single-exponential decay for the BD → pore transition. Data are relative to the RF(5)–(*E* ^1^(σ = 4) – *E* ^2^(σ = 4)) segmentation. Panels (a, b) show the comparison of τ_1_, τ_2_, and *a* when parts of HD are reattributed (in red) and are not (in blue), for the BD → pore and the BD → HD transitions, respectively. Panels (c, e) and (d, f) consider the case in which HD are not (respectively are) reclassified as BD. The box plots represent the 0.25–0.75 quartiles (with whiskers extending to 1.5 times the inner quartile range); in panels (c–f), the red dots are data outliers.

The configurations that yield the best exponential fit are also used to recalculate the transition from BD to HD (Figure 8b,d,f). As shown in Figure 8a, for all surfaces, the best fit was obtained by reclassifying part of the HD regions into the BD domain. The specific threshold used for reclassification (i.e., the residence time below which the HD regions are reclassified into BD) and the corresponding fitting errors are summarized in Table 2. Reclassification results in larger median values for both τ_1_ (around 60 ps, hence, approximately three times higher than the initial value before reclassification) and *a* (with around 90% of the population following τ_1_). Also for the BD → HD transitions, reclassification yields decays with larger median values of τ_1_ and *a* (Figure 8b).

**Table 2:** Residence-Time Constants (Transitions from BD) for the RF(5)–*E* ^1^(*σ* = 4) – *E* ^2^(*σ* = 4) Segmentation When Part of the HD Regions Are Reclassified as BD to Obtain a Closer Fit to a Single Exponential Decay for the BD → Pore^a^

		transition BD → pore				transition BD → HD			
SURF_ID_	HD_off_ (% *t*_tot_)	τ̅ (ps)	*a*	*r* ^2^	*N*_trans_ (%)	τ̅ (ps)	*a*	*r* ^2^	*N*_trans_ (%)
S1	2.9	41.72 (5.92)	0.94 (0.06)	0.94	0.69	29.79 (1.9)	0.71 (0.04)	0.83	0.31
S2	6.6	43.3 (4.21)	0.91 (0.06)	0.94	0.67	29.57 (1.87)	0.71 (0.03)	0.86	0.33
S3	1.5	29.39 (4.62)	0.92 (0.09)	0.95	0.68	18.23 (1.48)	0.58 (0.04)	0.84	0.32
S4	2.9	41.8 (4.9)	0.91 (0.1)	0.96	0.76	31.8 (2.14)	0.76 (0.04)	0.82	0.24
S5	1.7	26.05 (3.62)	0.88 (0.1)	0.92	0.46	17.4 (1.63)	0.64 (0.06)	0.91	0.54
S6	6.2	48.56 (4.51)	0.92 (0.07)	0.95	0.77	32.75 (1.92)	0.71 (0.03)	0.79	0.23
S7	4.3	41.8 (7.75)	0.96 (0.04)	0.94	0.6	31.19 (2.32)	0.76 (0.03)	0.89	0.4
S8	6.0	48.33 (6.62)	0.95 (0.07)	0.96	0.79	36.84 (1.87)	0.72 (0.04)	0.77	0.21
S9	8.4	47.73 (5.98)	0.94 (0.04)	0.95	0.76	37.61 (2.11)	0.76 (0.03)	0.81	0.24
S10	0.6	17.59 (3.88)	0.88 (0.14)	0.89	0.33	12.63 (1.62)	0.63 (0.07)	0.92	0.67
S11	3.3	32.99 (4.89)	0.93 (0.06)	0.95	0.74	22.15 (1.66)	0.66 (0.04)	0.82	0.26
S12	3.0	33.0 (5.11)	0.94 (0.05)	0.94	0.59	23.33 (1.76)	0.69 (0.04)	0.88	0.41
S13	8.4	52.07 (3.77)	0.9 (0.06)	0.95	0.79	36.17 (1.9)	0.73 (0.03)	0.79	0.21
S14	7.7	53.66 (6.54)	0.95 (0.03)	0.95	0.84	41.25 (1.95)	0.74 (0.04)	0.76	0.16
S15	1.5	27.01 (3.94)	0.91 (0.06)	0.91	0.41	19.44 (1.66)	0.66 (0.05)	0.92	0.59
S16	7.7	47.5 (5.2)	0.93 (0.05)	0.95	0.78	33.62 (2.0)	0.74 (0.03)	0.79	0.22
S17	6.3	42.41 (3.86)	0.9 (0.05)	0.94	0.64	25.9 (1.58)	0.62 (0.04)	0.86	0.36
S18	1.0	22.56 (5.41)	0.93 (0.09)	0.92	0.42	16.81 (1.7)	0.67 (0.06)	0.93	0.58
S19	3.6	32.68 (3.49)	0.88 (0.07)	0.95	0.77	24.31 (2.01)	0.74 (0.04)	0.82	0.23
S20	9.8	54.58 (6.7)	0.96 (0.04)	0.96	0.82	39.42 (2.18)	0.74 (0.03)	0.78	0.18
S21	5.8	47.9 (6.61)	0.95 (0.04)	0.95	0.82	35.33 (2.0)	0.75 (0.04)	0.74	0.18
S22	4.8	44.61 (5.93)	0.94 (0.04)	0.96	0.78	33.57 (2.11)	0.76 (0.04)	0.81	0.22
S23	4.5	46.19 (6.44)	0.95 (0.04)	0.95	0.67	35.86 (2.37)	0.78 (0.03)	0.88	0.33
S24	4.4	45.33 (7.33)	0.96 (0.03)	0.95	0.78	30.92 (1.8)	0.7 (0.04)	0.8	0.22

a The HD_off_ quantifies (as % of the overall simulation time) up to which residence time the HD are reclassified as BD (i.e., all the HD regions in which molecules have overall spent up to that % of time), for each of the 24 surfaces. Table displays for the BD → pore and BD → HD transitions, the mean residence times (τ̅) as computed by means of eqs 1 and 2, the *r* ^2^ factor, and the number of transition events (*N*_trans_). The uncertainties on the fitted parameters, estimated as the standard deviation obtained from the covariance matrix, are given in parentheses.

Comparing the residence times of the initial segmentation (RF(5)) with those obtained after reclassification of HD regions (RF(5)^reclass^), we observe that the latter leads to a broader τ_1_ distribution for both transitions (see Figure 8a,b). Also the distributions of the mean residence time τ̅ widen, ranging from 17 to 53 ps and from 12 to 41 ps for the BD → pore and the BD → HD transitions, respectively (Table 2). The broadening depends on the fraction of HD regions that are reclassified as BD. In some cases (e.g., surface S10; see Table in Supporting Information), the *a* factors for different iterations are close, and more HD could be reclassified to BD without significantly degrading the single-exponential approximation of the residence-time distribution. This would increase some of the τ̅ values in Table 2 and reduce the spread of the distributions.

#### Transitions from HD to Pore and BD

We now examine the HD regions remaining after reclassification and analyze the residence time conditional on the transition from HD to the pore (HD → pore) and to the BD class (HD → BD). As detailed in the Methods, the total residence time of each molecule in each HD region is computed over 400 ps time windows. If a molecule temporarily exits (for a few frames) and then re-enters the same HD region, it is not considered desorbed. However, frames spent outside the HD region are excluded from the residence time. This prevents the attribution of residence time to the wrong class (i.e., HD instead of BD) and avoids double counting in both HD and BD statistics. Although this leads to a slight underestimation of the actual residence time, the effect is limited and does not significantly impact overall trends.

The residence-time distributions for both transitions are shown in Figure 9 for a reference surface, while the mean adsorption time (τ̅) for each surface is reported in Table 3. The HD → BD transition has a considerably higher frequency than the HD → pore (see *N*_trans_ in Table 3). This indicates that in most cases molecules are expected to move to the BD region before being desorbed from the adsorption layer, as is expected because of the lower energy of molecules in BD than in the pore. The relatively higher frequency of the HD → BD allows sufficient data to be fitted to eq 1. As shown in Table 3 and in Figure 9, the HD → BD transition is well described by a biexponential decay, which is the signature of the heterogeneity of the adsorption capacities of the HD regions. The τ̅ values on the 24 surfaces are widely distributed, ranging from 33 to 90 ps. The parameter *a* in Table 3 can be interpreted as a descriptor of the degree of heterogeneity, with its value approaching 1 when HD regions have more uniform adsorption capacities.

**Figure 9:**
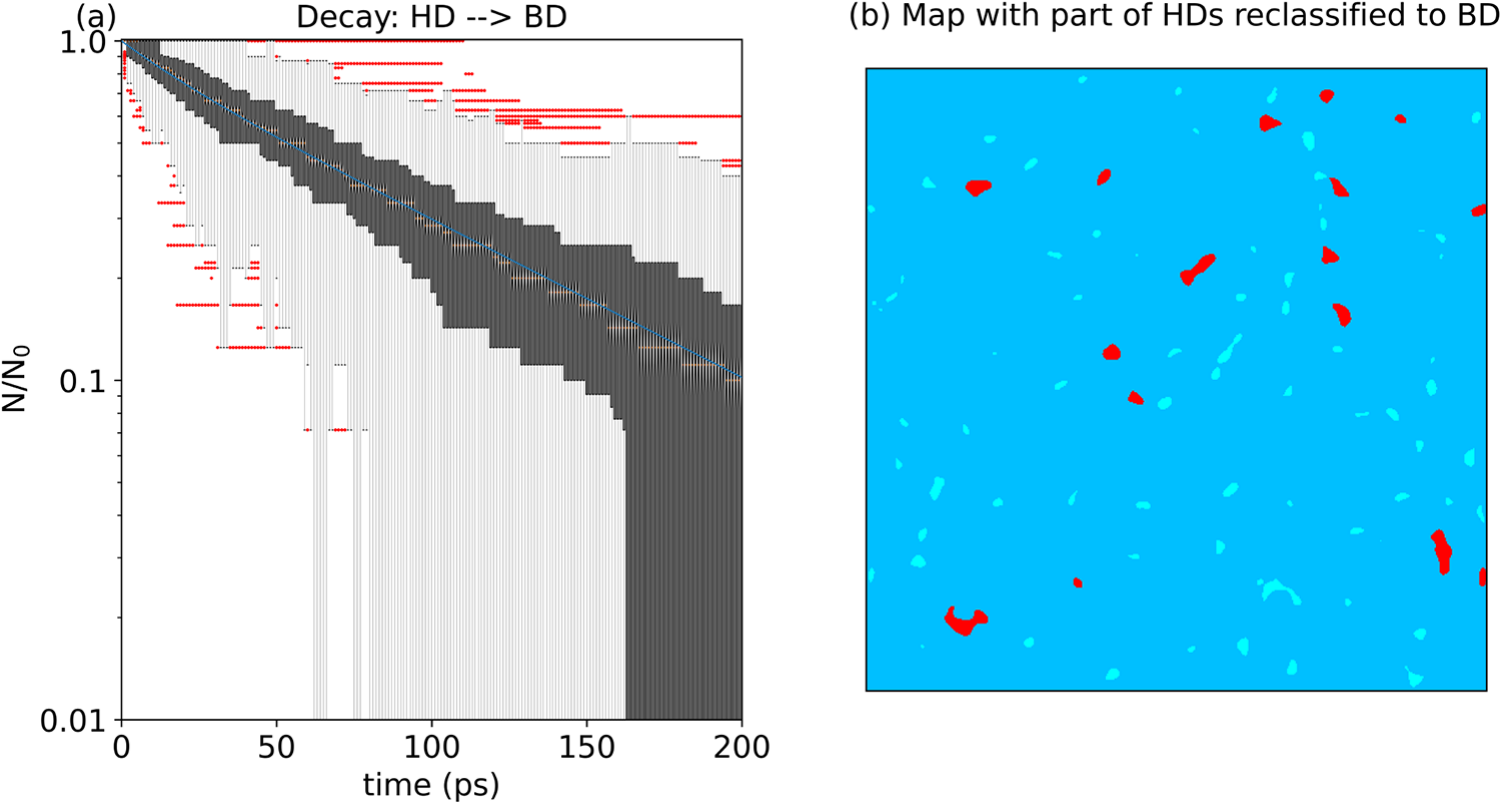
(a) Residence-time distributions (for a reference surface) for the HD → BD transition when part of the HD regions are reclassified as BD. The box plots represent the 0.25–0.75 quartiles (with whiskers extending to 1.5 times the inner quartile range). (b) Segmented density map with ID and LD classes merged into BD (in light-blue), part of the HD regions reclassified to BD (in cyan), and the remaining HD regions (in red).

**Table 3:** Residence-Time Constants (Transitions from HD) for the RF(5) −*E* ^1^(*σ* = 4) – *E* ^2^(*σ* = 4) Segmentation when Part of the HD Regions Are Reclassified as BD to Obtain a Closer Fit to a Single Exponential Decay for the BD → Pore^a^

		transition HD → B*D*				transition HD → pore	
SURF_ID_	HD_off_ (% *t*_tot_)	τ̅ (ps)	*a*	*r* ^2^	*N*_trans_ (%)	τ̅ (ps)	*N*_trans_ (%)
S1	2.9	79.44 (2.27)	0.77 (0.05)	0.82	0.9	NC	0.1
S2	6.6	55.11 (2.77)	0.57 (0.13)	0.85	0.96	NC	0.04
S3	1.5	70.62 (1.67)	0.56 (0.04)	0.79	0.83	NC	0.17
S4	2.9	90.24 (1.88)	0.71 (0.03)	0.7	0.84	NC	0.16
S5	1.7	58.23 (1.89)	0.53 (0.06)	0.88	0.95	NC	0.05
S6	6.2	83.65 (3.69)	0.86 (0.07)	0.81	0.84	NC	0.16
S7	4.3	69.82 (2.89)	0.55 (0.12)	0.84	0.89	NC	0.11
S8	6.0	64.15 (3.41)	0.5 (0.15)	0.73	0.89	NC	0.11
S9	8.4	81.08 (2.59)	0.73 (0.08)	0.8	0.81	NC	0.19
S10	0.6	47.81 (1.77)	0.54 (0.06)	0.9	0.94	NC	0.06
S11	3.3	64.5 (2.43)	0.67 (0.09)	0.79	0.9	NC	0.1
S12	3.0	51.5 (2.28)	0.73 (0.08)	0.88	0.96	NC	0.04
S13	8.4	82.55 (1.7)	0.64 (0.03)	0.65	0.82	NC	0.18
S14	7.7	70.0 (3.93)	0.91 (0.03)	0.68	0.96	NC	0.04
S15	1.5	33.97 (2.06)	0.6 (0.1)	0.87	0.97	NC	0.03
S16	7.7	49.17 (3.5)	0.58 (0.2)	0.74	0.97	NC	0.03
S17	6.3	55.38 (3.48)	0.4 (0.13)	0.87	0.88	NC	0.12
S18	1.0	51.84 (1.66)	0.53 (0.05)	0.84	0.96	NC	0.04
S19	3.6	58.38 (3.39)	0.34 (0.1)	0.8	0.85	NC	0.15
S20	9.8	59.12 (4.44)	0.88 (0.12)	0.79	0.97	NC	0.03
S21	5.8	73.08 (3.01)	0.66 (0.13)	0.68	0.84	NC	0.16
S22	4.8	48.16 (4.55)	0.37 (0.18)	0.74	0.97	NC	0.03
S23	4.5	44.96 (3.35)	0.38 (0.13)	0.75	0.88	NC	0.12
S24	4.4	74.21 (2.01)	0.73 (0.04)	0.72	0.96	NC	0.04

a The HD_off_ quantifies (as % of the overall simulation time) up to which residence time the HD are reclassified as BD (i.e., all the HD regions in which molecules have overall spent up to that % of time), for each of the 24 surfaces. Table displays, for the HD → BD and HD → pore transitions, the number of transition events (*N*_trans_). The mean residence times (τ̅) as computed by means of eqs 1 and 2 and the *r* ^2^ factor are given only for the HD → BD; for the HD → pore transition, values are not reported due to the scarcity of data in the fitting. The uncertainties on the fitted parameters, estimated as the standard deviation obtained from the covariance matrix, are given in parentheses.

For the HD → pore transition, the number of observed events is too limited to perform a robust statistical analysis. The few transitions that occurred are characterized by substantially larger time constants than those of the HD → BD transitions, approaching the duration of the time windows used in the analysis (i.e., 400 ps). This is due to the larger energy difference between the states, which makes direct desorption from HD to the pore a rare event. When implementing the microkinetic or KMC models of surface dynamics, the rare HD → pore transitions can be neglected, assuming that CO_2_ desorbs into the pore only from the BD domain.

### Conclusions

We introduced a modified RF classifier to segment surface density maps into regions with distinct adsorptivity. The robustness of the classifier was enhanced by (i) reducing the feature set from 16 to 4 to avoid redundancy and inflation of feature importance that may arise when the same feature is considered multiple times;^28^ (ii) standardizing the cutoffs used to define the training domains across all smoothing levels and surfaces, improving consistency across segmentations. The selection of training data is based on mathematical criteria (specifically, the sign of the two Hessian eigenvalues) rather than on visual inspection, avoiding biases associated with manual labeling.^39,40^ The new protocol enables systematic control over the morphology and extent of segmented classes via smoothing parameters while minimizing classification uncertainty.

The proposed framework is flexible, robust, and suitable for a wide range of classification problems. Here, we applied the RF classifier to adsorption density maps generated from extensive MD simulations of CO_2_ confined within amorphous silica slit pores. These maps reveal distinct adsorption regions corresponding to varying local residence times of the CO_2_ molecules. The segmentation protocol was optimized to support kinetic interpretability, identifying heterogeneous HD regions that retain adsorbate molecules for extended times and maximizing the homogeneous regions where desorption follows exponential kinetics. To this end, low- and intermediate-density regions were merged into a background-density (BD) class, and portions of the HD regions were recursively reclassified into the BD class to improve the exponentiality of the BD residence-time distribution. The final segmentation was selected by maximizing the quality of the fit. Across all surfaces, reclassification improves fitting, demonstrating that only a small fraction of HD regions drives heterogeneous adsorption, with biexponential fits revealing the presence of multiple time scales.

After reclassification, we analyzed residence-time kinetics by extracting the residence times conditional on all possible transitions: between BD and HD, as well as from BD and HD to the pore. The observed nonexponential behavior indicates time-dependent transition probabilities and potential memory effects. Under these conditions, the relationship between residence-time distributions and transition rates becomes nontrivial, as effective rate constants cannot be simply derived from the inverse of the characteristic times. Instead, explicit assumptions about the mesoscale kinetics are required. In this work, we focused on extracting residence-time statistics, deferring full rate inference to future studies. These statistics can inform upscaled microkinetic or kinetic Monte Carlo models, providing a computationally efficient framework to simulate adsorption dynamics over larger domains and longer times^32,33^ that can be related to experimental data.^41^

The direct transition from HD to the pore was rare, reflecting strong binding at uncoordinated defects (Si3 and NBO), and the residence times often exceeded the observation window. As the large energy difference makes direct desorption unlikely, molecules in HD regions tend to follow a two-step desorption pathway, first transitioning to the intermediate-energy BD state and subsequently desorbing from the BD class to the pore. This two-step mechanism justifies neglecting the direct transition from the HD regions to the pore in upscaled models.

Strong adsorption in HD regions is attributed to undercoordinated defects (Si3 and NBO) and hints the potential role of reactive surface defects, which could chemically bind the adsorbate, be exploited as an anchoring site for dopants, or participate in catalytic conversion with cosorbed species. While nonreactive MD captures physisorption, reactive events such as chemisorption require higher-accuracy approaches (DFT, ab initio MD, or reactive force fields, including machine learning interatomic potentials) to provide kinetic constants for mesoscale modeling.

## Supplementary Material



## Data Availability

All software used in the work is open source. All data generated in this work are available on the following GitHub repository: https://github.com/mattitur/opt_seg.

## Notes

Feature importance analysis (Figure S1); surface density of surface groups (Table S1); features correlation (Figure S2); decay constants for different smoothing combinations (Tables S2–S7); reclassification of HD regions for one reference surface (Table S8) (PDF)
