# Supplementary material for: Segmentation for Learning Adsorption Patterns and Residence-Time Kinetics on Amorphous Surfaces
Source: J Chem Inf Model. 2025 Oct 3;65(20):10903–17. doi: 10.1021/acs.jcim.5c01463 (PMC12570132; doi:10.1021/acs.jcim.5c01463)
Supplement: Supplementary file 1 [file ci5c01463_si_001.pdf]

Supporting Information: Segmentation for Learning Adsorption Patterns and Residence-Time Kinetics on Amorphous Surfaces

Mattia Turchi* and Ivan Lunati

Laboratory for Computational Engineering, Swiss Federal Laboratories for Materials Science and Technology, Empa, 8600 Dübendorf, Switzerland

E-mail: mattia.turchi@empa.ch

Creation of defective amorphous silica surfaces and surface sites densities

The slit nanopores are formed by two slabs which extend for around 100 Å × 100 Å × 38 Å in x, y and z directions, respectively. The width of the pores (in the z direction) is 2 nm. Periodic boundary conditions are applied in all three directions. This pore size has been used both for amorphous[1] and crystalline silica pores interacting with CO_2.[2] We remark that molecules are not affected by the silica surface if they are at a distance larger than 0.5 nm, and the concentration of the CO_2 molecules reaches a constant bulk value in the center of the pore.

To take into account the statistical variability of the amorphous pores we generate 12 different samples (i.e., 24 surfaces). The samples were generated in a previous work[3] using

a melt & quench procedure by means of MD simulations.[4–7] The followed protocol is the one proposed by Du et al. which uses the Buckingham potential[1,8] (see Turchi et al.[9] for the detailed description of the generation of the amorphous surfaces). In brief, the following steps are performed: (i) a bulk structure undergoes the melt & quench (T = 6000K, quencing rate = 10 K/ps), (ii) a vacuum gap is added to create the slit pore, (iii) the central part of the structure is frozen to keep the bulk-like structure while the layers close to the vacuum gap are re-annealed at a lower temperature to cure the surface defects. Nevertheless, after re-annealing a fraction of undercoordinated defects are still present at the surface (namely, undercoordinated or non-bridging oxygen, NBO; and undercoordinated silicon, Si3).

Next, the amorphous surfaces are hydroxylated by means of an *in-house* code which follows the protocol[1,9,10] to hydroxylate (resp. hydrogenate) all exposed Si3 (resp. NBO) sites which result from the melt & quench simulation, as well as the 2 and 3-membered rings (identified according to the Guttman[11] definition) which are broken to allow for the grafting of the OH groups. After hydroxylation, the surfaces are equilibrated for 1 ns in the NVT ensemble using the ClayFF potential,[12] which is later employed to simulate the CO_2/silica interface.

Some of the undercoordinated defects cannot be functionalized due to the surface roughness which renders some sites inaccessible to water molecules and prevents hydrogenation or hydroxylation.[9] Although each surface has different surface densities of Si3, NBO, and OH groups, the values are within the range of observed experimental data (see Table S1). In particular, the hydroxyl surface density is in the range of the experimental values reported by Zhuravlev et al.[13] and the concentration of surface defects (i.e., Si3 and NBO) agrees with those obtained by reactive MD simulations[14] or measured by infra-red spectroscopy.[15,16]

Table S1: Fraction of different groups at the surface.

$SURF_{ID}$	OH/nm^2	Si3/nm^2	NBO/nm^2
S1	1.8	0.24	0.3
S2	1.5	0.19	0.2
S3	1.6	0.21	0.2
S4	2.0	0.10	0.1
S5	1.7	0.25	0.2
S6	1.6	0.12	0.2
S7	2.0	0.23	0.2
S8	1.7	0.16	0.1
S9	2.2	0.25	0.27
S10	2.2	0.20	0.17
S11	2.2	0.26	0.32
S12	2.2	0.24	0.26
S13	2.4	0.29	0.24
S14	2.3	0.17	0.29
S15	2.2	0.29	0.18
S16	2.1	0.26	0.28
S17	2.8	0.32	0.21
S18	2.9	0.26	0.46
S19	2.7	0.25	0.37
S20	3.0	0.35	0.24
S21	2.8	0.29	0.22
S22	2.9	0.27	0.35
S23	2.7	0.19	0.18
S24	2.8	0.24	0.23

Details on MD simulations of CO_2/silica interactions

ClayFF[12] was employed to describe the hydroxylated silica and the dynamics and surface bonding of the CO_2 molecules, which were described by a flexible version of the EPM2 model.[17,18] For the SiO_2/CO_2 cross interactions terms we employed the parameters optimized and validated by Crabtree et al.[19] and Purton et al.[20] All parameters used in the ClayFF and EPM2 FF can be found in Turchi et al.[9] The reliability of ClayFF to treat amorphous silica has been demonstrated by several works. Bourg et al.[21] reported that ClayFF yields hydroxylated amorphous silica structures in agreement with experimental

data at room temperature.[22,23] Leroch et al.[24] showed that ClayFF provides an accurate description of humid amorphous silica surfaces and yields water adsorption isotherms for hydroxylated amorphous silica pores which agree very well with experimental data.[25] The suitability of ClayFF was also shown for simulation of partially hydroxylated silica surfaces[26]

All simulations were performed in the NVT ensemble with a time step of 1 fs. Two separated Nosè-Hoover[27,28] thermostats, with a relaxation time of 100 fs, keep the temperatures of the CO_2 and the silica substrate at 290 K. Each simulation was run for 100 ns of which the last 90 ns are used for analyzing results. A cutoff of 10 Å was employed for the short-range Van der Waals as well as for the electrostatic interactions. The long range coulombic interactions were treated with the staggered 3Particle–Mesh (PPPM) method,[29–31] with an accuracy of 10^{-4}.

Features reduction

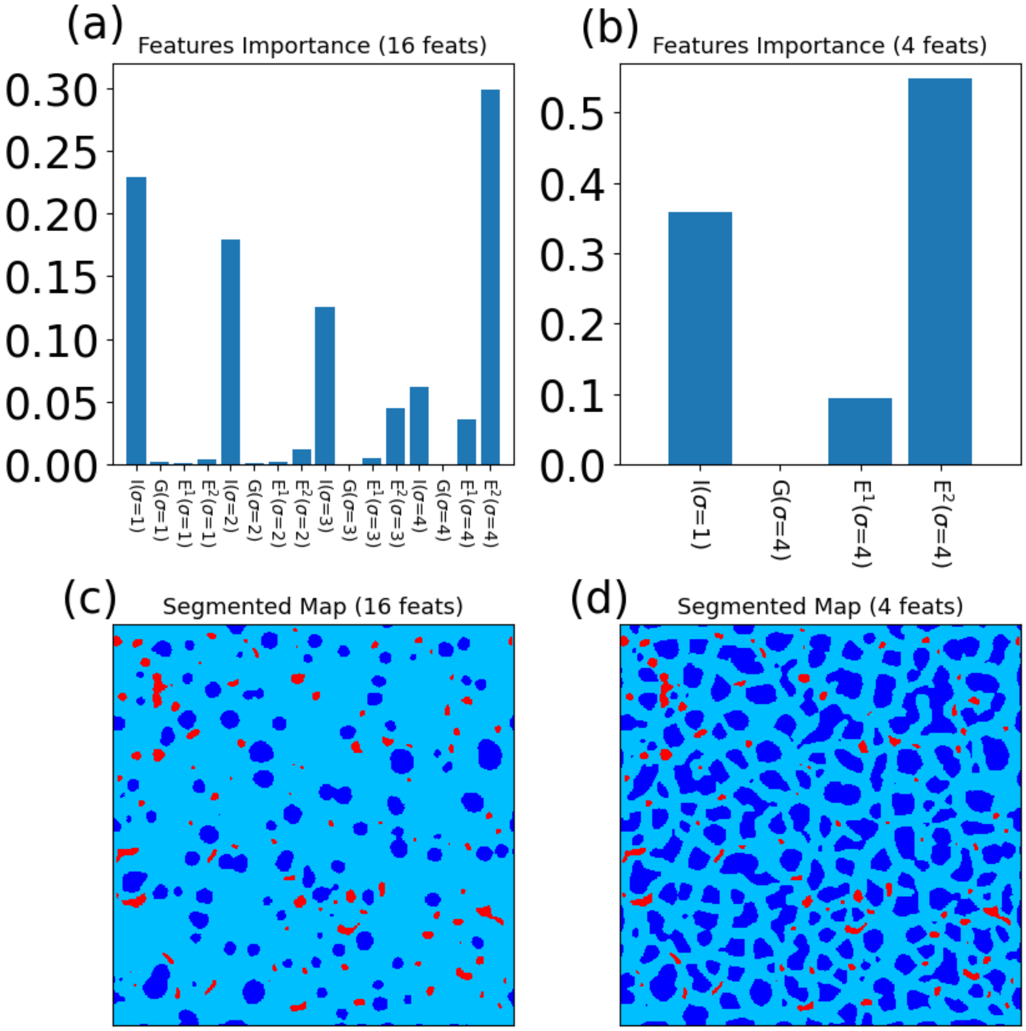

Figure S1: Features importance for the 16- (panel a) resp. 4-features (panel b) and segmented maps for the 16- (panel c) resp. 4-features (panel d) segmentations for one reference surface.

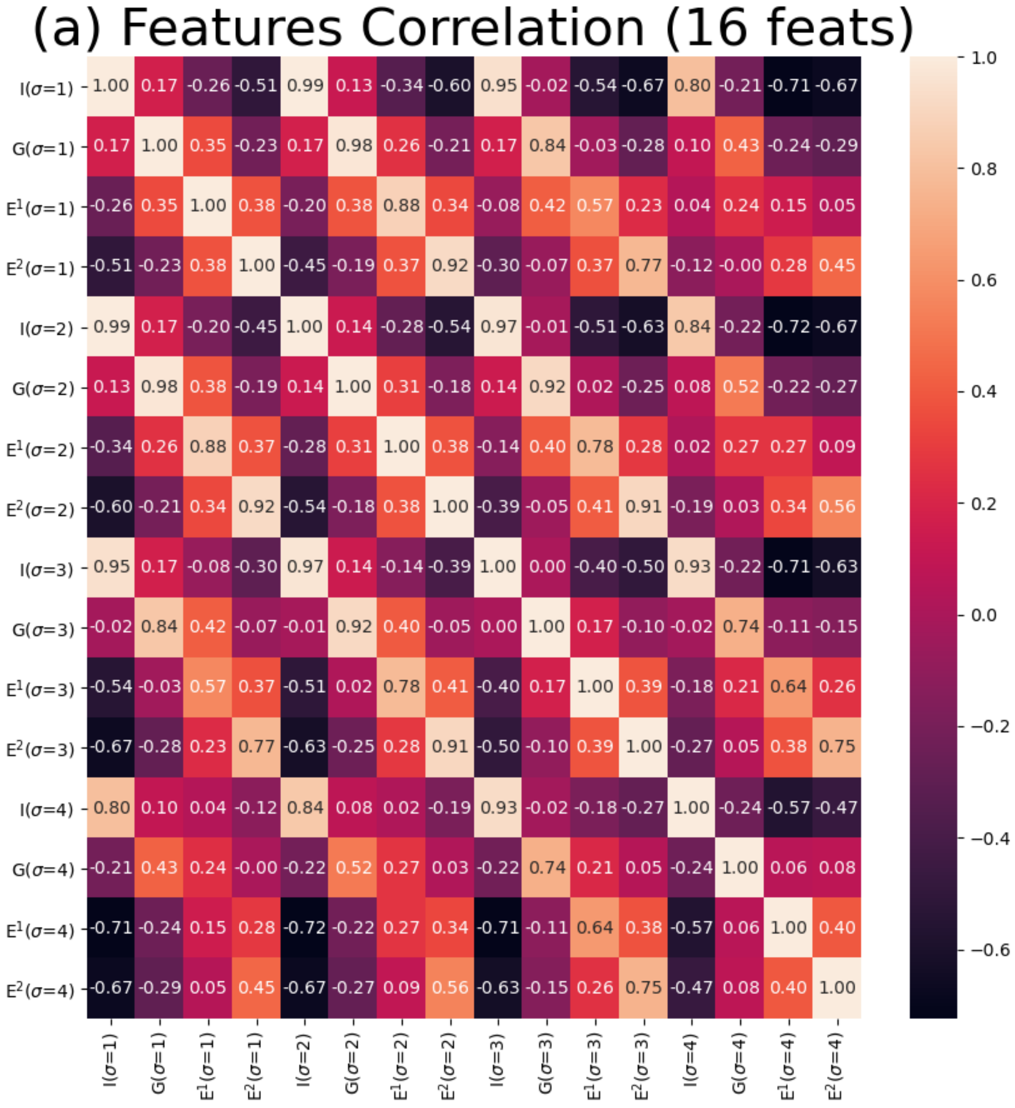
(a) Features Correlation (16 feats)

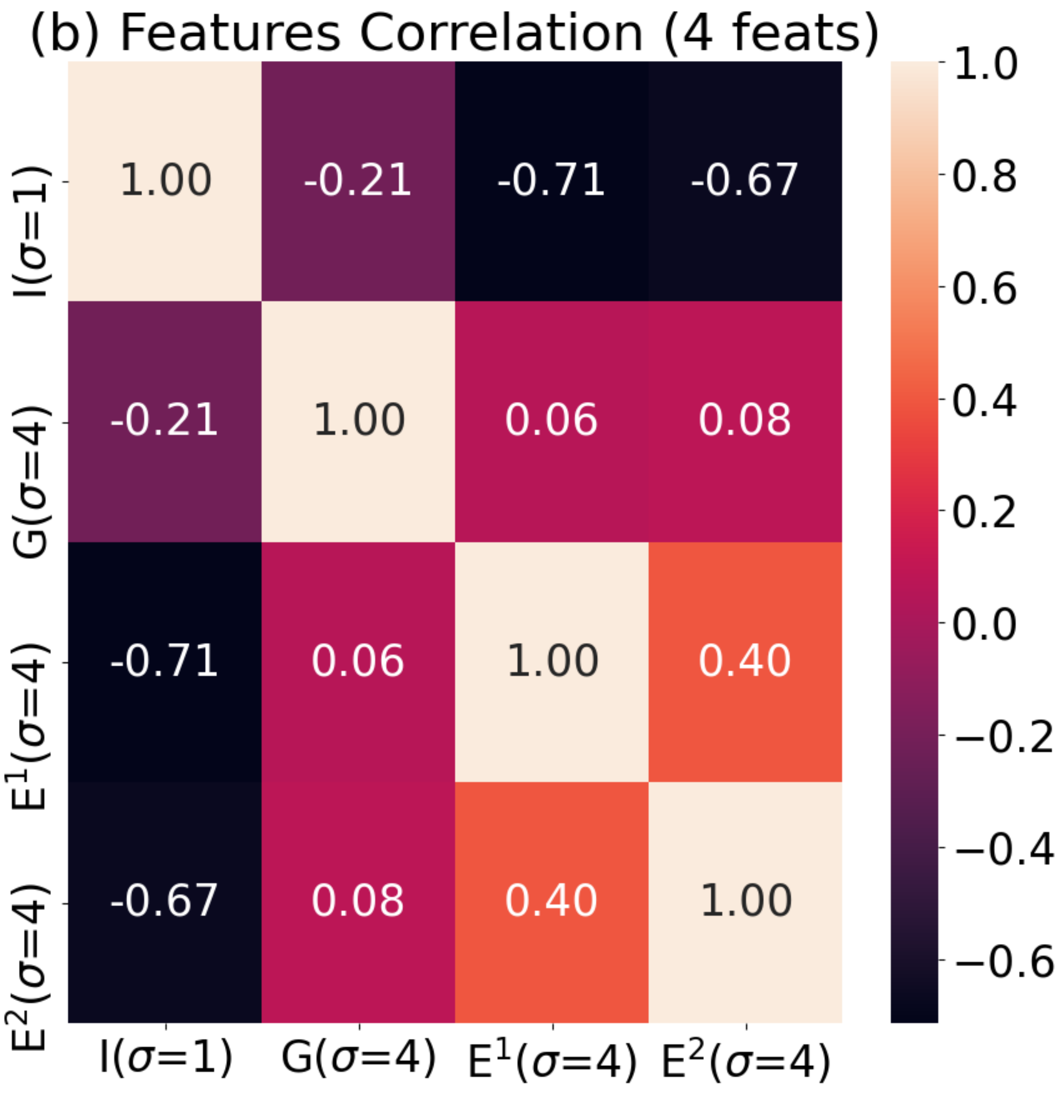
(b) Features Correlation (4 feats)
I(σ=1)
G(σ=4)
E¹(σ=4)
E²(σ=4)
1.00
-0.21
-0.71
-0.67
-0.21
1.00
0.06
0.08
-0.71
0.06
1.00
0.40
-0.67
0.08
0.40
1.00
1.0
0.8
0.6
0.4
0.2
0.0
−0.2
−0.4
−0.6

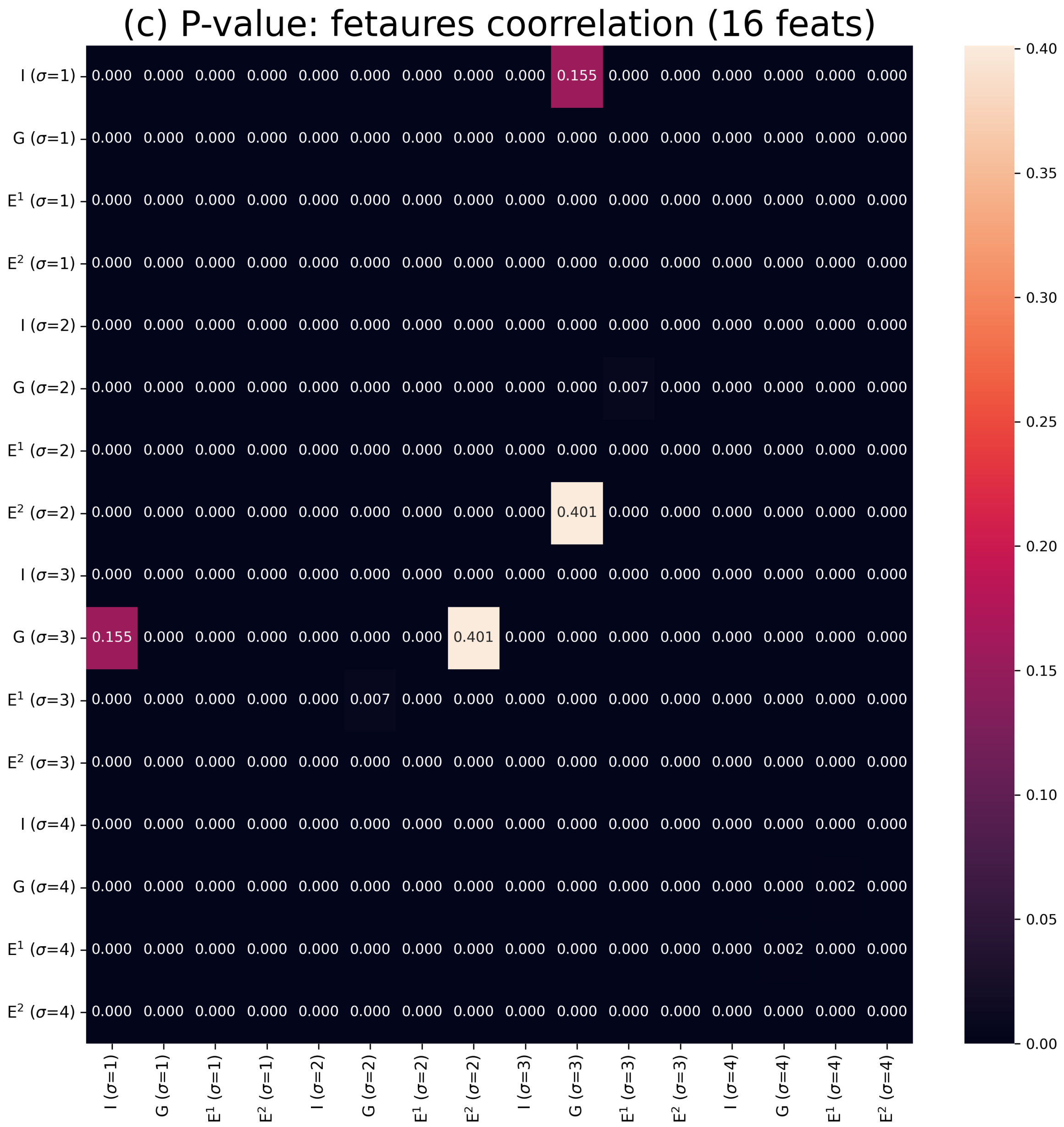
(c) P-value: fetaures coorrelation (16 feats)
I (σ=1) 0.000 0.000 0.000 0.000 0.000 0.000 0.000 0.000 0.000 0.155 0.000 0.000 0.000 0.000 0.000 0.000
G (σ=1) 0.000 0.000 0.000 0.000 0.000 0.000 0.000 0.000 0.000 0.000 0.000 0.000 0.000 0.000 0.000 0.000
E^1 (σ=1) 0.000 0.000 0.000 0.000 0.000 0.000 0.000 0.000 0.000 0.000 0.000 0.000 0.000 0.000 0.000 0.000
E^2 (σ=1) 0.000 0.000 0.000 0.000 0.000 0.000 0.000 0.000 0.000 0.000 0.000 0.000 0.000 0.000 0.000 0.000
I (σ=2) 0.000 0.000 0.000 0.000 0.000 0.000 0.000 0.000 0.000 0.000 0.000 0.000 0.000 0.000 0.000 0.000
G (σ=2) 0.000 0.000 0.000 0.000 0.000 0.000 0.000 0.000 0.000 0.000 0.007 0.000 0.000 0.000 0.000 0.000
E^1 (σ=2) 0.000 0.000 0.000 0.000 0.000 0.000 0.000 0.000 0.000 0.000 0.000 0.000 0.000 0.000 0.000 0.000
E^2 (σ=2) 0.000 0.000 0.000 0.000 0.000 0.000 0.000 0.000 0.000 0.401 0.000 0.000 0.000 0.000 0.000 0.000
I (σ=3) 0.000 0.000 0.000 0.000 0.000 0.000 0.000 0.000 0.000 0.000 0.000 0.000 0.000 0.000 0.000 0.000
G (σ=3) 0.155 0.000 0.000 0.000 0.000 0.000 0.000 0.401 0.000 0.000 0.000 0.000 0.000 0.000 0.000 0.000
E^1 (σ=3) 0.000 0.000 0.000 0.000 0.000 0.007 0.000 0.000 0.000 0.000 0.000 0.000 0.000 0.000 0.000 0.000
E^2 (σ=3) 0.000 0.000 0.000 0.000 0.000 0.000 0.000 0.000 0.000 0.000 0.000 0.000 0.000 0.000 0.000 0.000
I (σ=4) 0.000 0.000 0.000 0.000 0.000 0.000 0.000 0.000 0.000 0.000 0.000 0.000 0.000 0.000 0.000 0.000
G (σ=4) 0.000 0.000 0.000 0.000 0.000 0.000 0.000 0.000 0.000 0.000 0.000 0.000 0.000 0.000 0.002 0.000
E^1 (σ=4) 0.000 0.000 0.000 0.000 0.000 0.000 0.000 0.000 0.000 0.000 0.000 0.000 0.000 0.002 0.000 0.000
E^2 (σ=4) 0.000 0.000 0.000 0.000 0.000 0.000 0.000 0.000 0.000 0.000 0.000 0.000 0.000 0.000 0.000 0.000
I (σ=1) G (σ=1) E^1 (σ=1) E^2 (σ=1) I (σ=2) G (σ=2) E^1 (σ=2) E^2 (σ=2) I (σ=3) G (σ=3) E^1 (σ=3) E^2 (σ=3) I (σ=4) G (σ=4) E^1 (σ=4) E^2 (σ=4)
0.40
0.35
0.30
0.25
0.20
0.15
0.10
0.05
0.00

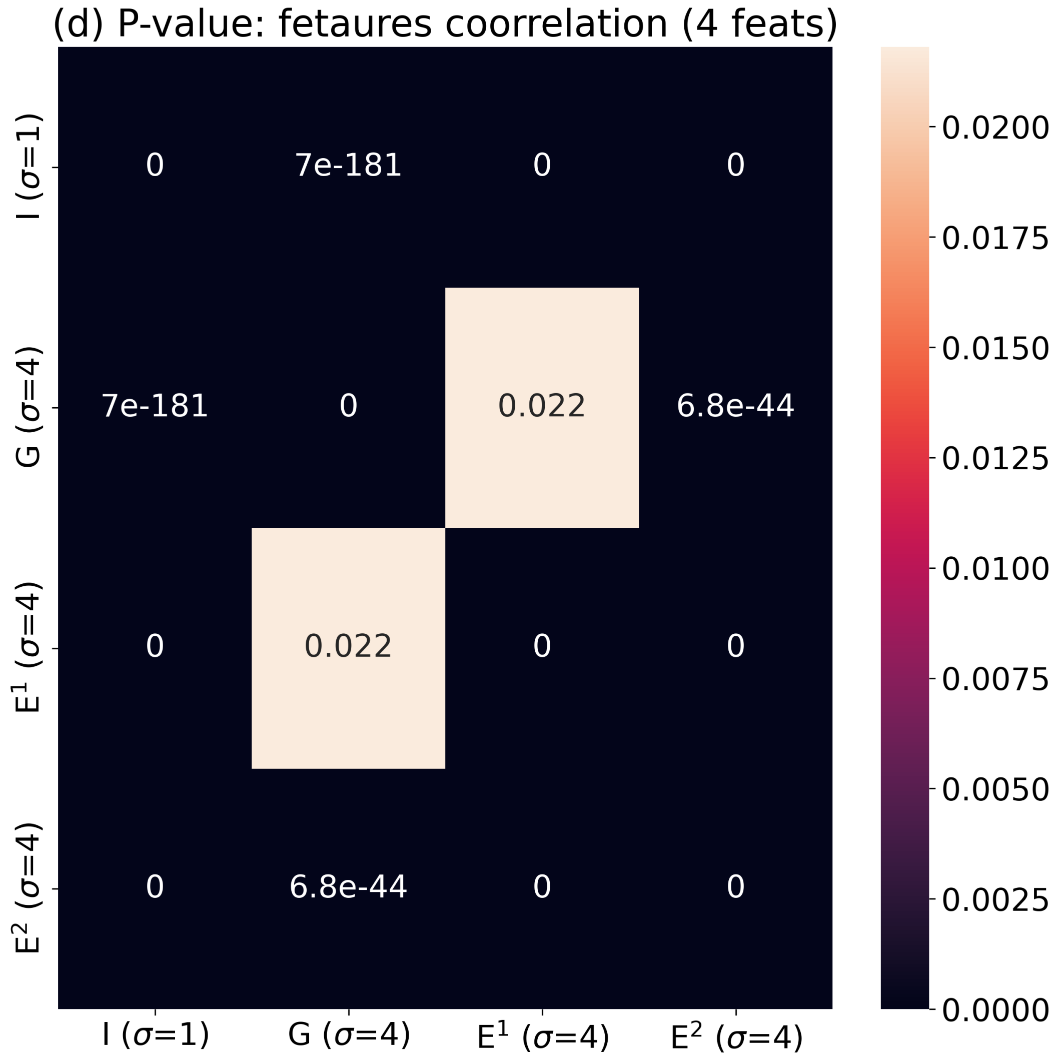

Figure S2: Features correlations for the 16- (panel a) resp. 4-features (panel b) segmentations for one reference surface. P-value relative to feature correlations for 16- (panel c) resp. 4-features (panel d) segmentations for the same reference surface.

The Spearman's rank coefficient between each pair of features is displayed in Figure 2a and 2b for the 16-features and 4-features segmentation, respectively. The p-values associated to each Spearman's coefficient are displayed in Figure 2c and 2d for the 16-features and 4-features segmentation, respectively. For the p-values, coefficients below 0.05 suggest that

the Spearman's coefficients, calculated on the randomly selected training data, are unlikely to have occurred by chance (i.e., there is less than 5 % chance that the observed correlation occurred by chance). In the 16-features segmentation, two combinations lead to coefficients higher than 0.05 while the rest of the values are close to 0. In the 4-features segmentation (the setup used in this manuscript) the G-E^1 combination has a value of 0.02, which, although higher than for other pairs, is still below 0.05.

Decay constants for smoothing combination: $E^1(\sigma = 4) - E^2(\sigma = 4)$

RF(5)

Table S2: Decays constants for the transition from the background for the $RF(5)$ segmentation and the $E^1(\sigma = 4) - E^2(\sigma = 4)$ smoothing.

		Transition $BD \to pore$				Transition $BD \to HD$			
$SURF_{ID}$	HD_{off} (% t_{tot})	$\bar{\tau}$ (ps)	a	r^2	N_{trans} (%)	$\bar{\tau}$ (ps)	a	r^2	N_{trans} (%)
S1	0.0	19.09 (3.66)	0.86 (0.15)	0.88	0.5	27.78 (1.76)	0.69 (0.04)	0.84	0.5
S2	0.0	16.02 (4.18)	0.62 (0.48)	0.88	0.38	19.84 (1.62)	0.64 (0.04)	0.9	0.62
S3	0.0	16.07 (3.67)	0.87 (0.16)	0.91	0.68	27.8 (1.78)	0.67 (0.03)	0.72	0.32
S4	0.0	16.0 (3.89)	0.81 (0.32)	0.9	0.43	21.47 (1.88)	0.73 (0.05)	0.91	0.57
S5	0.0	15.4 (3.97)	0.86 (0.21)	0.89	0.45	28.87 (1.76)	0.69 (0.04)	0.85	0.55
S6	0.0	16.63 (3.84)	0.71 (0.41)	0.87	0.41	19.83 (1.64)	0.65 (0.05)	0.89	0.59
S7	0.0	19.21 (3.33)	0.69 (0.3)	0.87	0.38	27.29 (1.99)	0.73 (0.03)	0.89	0.62
S8	0.0	18.65 (3.1)	0.76 (0.23)	0.9	0.39	17.05 (1.69)	0.66 (0.06)	0.91	0.61
S9	0.0	20.48 (2.91)	0.84 (0.11)	0.9	0.45	20.98 (1.77)	0.7 (0.05)	0.89	0.55
S10	0.0	14.57 (3.49)	0.85 (0.2)	0.88	0.61	52.61 (2.78)	0.81 (0.02)	0.72	0.39
S11	0.0	14.95 (2.92)	0.83 (0.14)	0.91	0.57	21.84 (1.66)	0.66 (0.04)	0.83	0.43
S12	0.0	15.77 (3.18)	0.75 (0.27)	0.88	0.45	26.61 (1.83)	0.71 (0.04)	0.87	0.55
S13	0.0	15.58 (2.91)	0.82 (0.15)	0.88	0.36	17.53 (1.8)	0.7 (0.06)	0.92	0.64
S14	0.0	16.84 (2.83)	0.81 (0.14)	0.88	0.37	15.9 (1.8)	0.7 (0.07)	0.92	0.63
S15	0.0	14.44 (3.35)	0.82 (0.22)	0.86	0.29	16.22 (1.57)	0.62 (0.06)	0.92	0.71
S16	0.0	19.88 (3.19)	0.84 (0.14)	0.9	0.44	19.58 (1.74)	0.68 (0.06)	0.89	0.56
S17	0.0	15.88 (2.72)	0.81 (0.14)	0.88	0.39	20.34 (1.59)	0.62 (0.05)	0.89	0.61
S18	0.0	14.1 (5.67)	0.93 (0.15)	0.9	0.49	37.64 (1.78)	0.7 (0.04)	0.82	0.51
S19	0.0	13.91 (2.86)	0.83 (0.13)	0.91	0.47	13.62 (1.72)	0.68 (0.07)	0.91	0.53
S20	0.0	17.48 (3.14)	0.83 (0.16)	0.91	0.38	19.37 (1.85)	0.71 (0.05)	0.92	0.62
S21	0.0	17.28 (3.44)	0.88 (0.11)	0.9	0.42	18.75 (1.72)	0.67 (0.06)	0.89	0.58
S22	0.0	16.58 (3.4)	0.75 (0.3)	0.9	0.38	13.73 (1.68)	0.62 (0.09)	0.93	0.62
S23	0.0	16.29 (3.78)	0.82 (0.28)	0.88	0.31	15.08 (1.76)	0.68 (0.07)	0.94	0.69
S24	0.0	20.3 (4.22)	0.91 (0.06)	0.92	0.47	21.12 (1.73)	0.68 (0.06)	0.9	0.53

RF(3)

Table S3: Decays constants for the transition from the background for the $RF(3)$ segmentation and the $E^1(\sigma = 4) - E^2(\sigma = 4)$ smoothing.

		Transition $BD{\rightarrow}pore$				Transition $BD{\rightarrow}HD$			
$SURF_{ID}$	HD_{off} (% t_{tot})	$\bar{\tau}$ (ps)	a	r^2	N_{trans} (%)	$\bar{\tau}$ (ps)	a	r^2	N_{trans} (%)
S1	0.0	7.2 (2.85)	0.69 (0.37)	0.74	0.2	5.15 (2.28)	0.65 (0.3)	0.94	0.8
S2	0.0	5.82 (5.77)	0.36 (0.67)	0.74	0.19	5.06 (2.29)	0.57 (0.31)	0.94	0.81
S3	0.0	5.85 (5.23)	0.46 (0.8)	0.8	0.25	4.78 (2.06)	0.66 (0.24)	0.93	0.75
S4	0.0	5.64 (4.77)	0.62 (0.97)	0.76	0.21	4.53 (2.39)	0.69 (0.32)	0.94	0.79
S5	0.0	6.04 (2.56)	0.82 (0.15)	0.75	0.2	4.67 (2.0)	0.62 (0.24)	0.93	0.8
S6	0.0	5.6 (3.16)	0.53 (0.48)	0.73	0.19	4.65 (2.44)	0.73 (0.29)	0.94	0.81
S7	0.0	8.16 (5.69)	0.94 (0.1)	0.79	0.18	6.75 (1.91)	0.62 (0.18)	0.95	0.82
S8	0.0	2.78 (25.65)	0.34 (3.8)	0.78	0.19	4.74 (2.28)	0.61 (0.32)	0.94	0.81
S9	0.0	5.52 (7.99)	0.15 (0.33)	0.77	0.2	5.02 (2.21)	0.6 (0.29)	0.94	0.8
S10	0.0	5.91 (2.79)	0.69 (0.4)	0.74	0.19	4.87 (2.0)	0.62 (0.23)	0.93	0.81
S11	0.0	5.08 (2.6)	0.66 (0.39)	0.78	0.23	4.3 (2.17)	0.65 (0.28)	0.94	0.77
S12	0.0	6.08 (2.73)	0.6 (0.39)	0.73	0.21	4.65 (2.21)	0.58 (0.3)	0.94	0.79
S13	0.0	5.96 (2.52)	0.69 (0.32)	0.75	0.21	5.07 (2.58)	0.57 (0.38)	0.94	0.79
S14	0.0	6.19 (2.5)	0.69 (0.31)	0.76	0.21	5.85 (2.08)	0.68 (0.21)	0.93	0.79
S15	0.0	4.97 (4.29)	0.59 (0.86)	0.72	0.16	4.31 (2.42)	0.7 (0.34)	0.94	0.84
S16	0.0	6.66 (2.45)	0.79 (0.18)	0.77	0.22	5.7 (2.03)	0.59 (0.23)	0.93	0.78
S17	0.0	4.76 (6.57)	0.43 (1.08)	0.77	0.18	4.48 (2.26)	0.64 (0.31)	0.94	0.82
S18	0.0	5.5 (3.17)	0.69 (0.52)	0.73	0.18	4.38 (2.32)	0.6 (0.34)	0.94	0.82
S19	0.0	5.68 (2.39)	0.62 (0.32)	0.82	0.27	4.71 (2.18)	0.6 (0.29)	0.94	0.73
S20	0.0	5.87 (4.81)	0.46 (0.72)	0.79	0.19	4.65 (2.28)	0.6 (0.32)	0.95	0.81
S21	0.0	5.71 (2.95)	0.83 (0.22)	0.76	0.2	5.18 (2.19)	0.68 (0.25)	0.94	0.8
S22	0.0	5.5 (9.67)	0.5 (1.9)	0.78	0.2	4.77 (2.41)	0.7 (0.31)	0.94	0.8
S23	0.0	5.57 (3.38)	0.82 (0.37)	0.75	0.18	5.16 (2.15)	0.69 (0.24)	0.94	0.82
S24	0.0	6.23 (3.54)	0.68 (0.6)	0.8	0.21	5.4 (2.43)	0.78 (0.19)	0.95	0.79

THR

Table S4: Decays constants for the transition from the background for the THR segmentation and the $E^1(\sigma = 4) - E^2(\sigma = 4)$ smoothing.

		Transition $BD\rightarrow pore$				Transition $BD\rightarrow HD$			
$SURF_{ID}$	HD_{off} (% t_{tot})	$\bar{\tau}$ (ps)	a	r^2	N_{trans} (%)	$\bar{\tau}$ (ps)	a	r^2	N_{trans} (%)
S1	0.0	9.86 (3.04)	0.46 (0.3)	0.78	0.2	7.97 (2.02)	0.48 (0.18)	0.94	0.8
S2	0.0	8.25 (3.21)	0.63 (0.44)	0.8	0.19	6.63 (1.87)	0.57 (0.18)	0.95	0.81
S3	0.0	9.17 (4.56)	0.21 (0.2)	0.77	0.24	6.25 (1.95)	0.61 (0.2)	0.94	0.76
S4	0.0	9.38 (3.96)	0.35 (0.32)	0.78	0.2	7.67 (2.54)	0.44 (0.25)	0.95	0.8
S5	0.0	14.73 (26.14)	0.01 (0.01)	0.57	0.18	6.99 (1.98)	0.56 (0.2)	0.94	0.82
S6	0.0	23.04 (7.89)	0.03 (0.01)	0.57	0.19	9.55 (3.08)	0.1 (0.04)	0.92	0.81
S7	0.0	10.84 (4.74)	0.4 (0.44)	0.78	0.18	8.25 (1.88)	0.61 (0.16)	0.95	0.82
S8	0.0	8.94 (6.53)	0.52 (0.98)	0.81	0.21	7.01 (1.96)	0.67 (0.17)	0.95	0.79
S9	0.0	8.48 (4.15)	0.48 (0.52)	0.82	0.2	6.93 (1.85)	0.64 (0.16)	0.95	0.8
S10	0.0	44.71 (12.13)	0.03 (0.01)	0.54	0.18	6.8 (1.89)	0.63 (0.17)	0.96	0.82
S11	0.0	7.66 (3.28)	0.58 (0.46)	0.85	0.23	6.54 (2.05)	0.54 (0.22)	0.95	0.77
S12	0.0	9.2 (3.41)	0.75 (0.41)	0.84	0.2	6.72 (1.87)	0.61 (0.17)	0.95	0.8
S13	0.0	9.64 (3.71)	0.33 (0.27)	0.78	0.21	8.11 (2.02)	0.56 (0.19)	0.94	0.79
S14	0.0	8.39 (2.9)	0.64 (0.37)	0.8	0.2	7.43 (2.24)	0.56 (0.25)	0.94	0.8
S15	0.0	9.15 (5.28)	0.36 (0.48)	0.78	0.18	7.77 (1.95)	0.64 (0.17)	0.95	0.82
S16	0.0	10.0 (2.97)	0.58 (0.35)	0.82	0.22	7.65 (1.98)	0.56 (0.19)	0.95	0.78
S17	0.0	9.12 (2.52)	0.6 (0.28)	0.82	0.18	7.76 (1.9)	0.58 (0.17)	0.96	0.82
S18	0.0	10.98 (7.0)	0.09 (0.1)	0.78	0.19	11.69 (2.17)	0.15 (0.04)	0.93	0.81
S19	0.0	73.72 (6.33)	0.06 (0.01)	0.58	0.26	10.18 (2.9)	0.09 (0.03)	0.9	0.74
S20	0.0	8.8 (2.86)	0.55 (0.34)	0.81	0.2	6.85 (1.91)	0.64 (0.18)	0.96	0.8
S21	0.0	9.78 (3.05)	0.6 (0.37)	0.78	0.23	7.85 (2.24)	0.74 (0.18)	0.94	0.77
S22	0.0	9.99 (3.82)	0.58 (0.51)	0.85	0.24	7.96 (1.94)	0.58 (0.18)	0.94	0.76
S23	0.0	9.5 (5.17)	0.54 (0.74)	0.82	0.19	7.89 (2.28)	0.74 (0.18)	0.96	0.81
S24	0.0	8.83 (2.84)	0.66 (0.34)	0.83	0.22	7.95 (2.11)	0.73 (0.16)	0.95	0.78

Decay constants for smoothing combination: $E^1(\sigma = 3) - E^2(\sigma = 4)$

RF(5)

Table S5: Decays constants for the transition from the background for the $RF(5)$ segmentation and the $E^1(\sigma = 3) - E^2(\sigma = 4)$ smoothing.

		Transition $BD \to pore$				Transition $BD \to HD$			
$SURF_{ID}$	HD_{off} (% t_{tot})	$\bar{\tau}$ (ps)	a	r^2	N_{trans} (%)	$\bar{\tau}$ (ps)	a	r^2	N_{trans} (%)
S1	0.0	3.45 (4.09)	0.75 (0.92)	0.7	0.15	15.99 (1.83)	0.71 (0.06)	0.91	0.85
S2	0.0	1.01 (0.26)	0.02 (0.0)	0.73	0.13	9.88 (1.66)	0.64 (0.09)	0.93	0.87
S3	0.0	16.07 (3.65)	0.87 (0.16)	0.91	0.68	27.83 (1.77)	0.67 (0.03)	0.72	0.32
S4	0.0	3.34 (12.63)	0.87 (8.94)	0.76	0.19	22.92 (1.92)	0.73 (0.05)	0.9	0.81
S5	0.0	2.53 (5.84)	0.62 (2.24)	0.7	0.15	16.83 (1.66)	0.66 (0.06)	0.9	0.85
S6	0.0	3.44 (3.71)	0.81 (0.58)	0.7	0.16	16.36 (1.56)	0.62 (0.06)	0.9	0.84
S7	0.0	3.52 (2.84)	0.79 (0.33)	0.63	0.13	20.41 (1.79)	0.7 (0.05)	0.91	0.87
S8	0.0	0.95 (1.34)	0.0 (0.0)	0.75	0.15	15.35 (1.63)	0.64 (0.06)	0.91	0.85
S9	0.0	14.83 (3.09)	0.86 (0.12)	0.89	0.39	20.28 (1.76)	0.7 (0.05)	0.9	0.61
S10	0.0	2.57 (7.38)	0.58 (2.56)	0.72	0.2	36.92 (1.85)	0.72 (0.03)	0.86	0.8
S11	0.0	14.98 (2.94)	0.83 (0.14)	0.91	0.56	22.36 (1.68)	0.66 (0.04)	0.83	0.44
S12	0.0	2.6 (11.33)	0.57 (3.9)	0.73	0.17	17.44 (1.62)	0.64 (0.06)	0.91	0.83
S13	0.0	4.16 (8.16)	0.83 (4.4)	0.77	0.16	11.19 (1.74)	0.67 (0.09)	0.94	0.84
S14	0.0	3.79 (5.89)	0.41 (1.0)	0.73	0.15	8.49 (1.72)	0.6 (0.13)	0.94	0.85
S15	0.0	3.86 (4.23)	0.83 (0.64)	0.68	0.12	8.58 (1.73)	0.59 (0.13)	0.94	0.88
S16	0.0	2.99 (5.03)	0.65 (1.88)	0.73	0.15	10.71 (1.62)	0.63 (0.08)	0.93	0.85
S17	0.0	15.79 (2.75)	0.81 (0.14)	0.88	0.39	20.82 (1.58)	0.62 (0.05)	0.89	0.61
S18	0.0	14.07 (5.73)	0.94 (0.14)	0.9	0.89	39.85 (2.1)	0.51 (0.02)	0.23	0.11
S19	0.0	13.92 (2.85)	0.83 (0.13)	0.91	0.47	13.63 (1.72)	0.68 (0.07)	0.91	0.53
S20	0.0	17.5 (3.12)	0.83 (0.16)	0.91	0.38	19.4 (1.85)	0.71 (0.05)	0.92	0.62
S21	0.0	17.2 (3.44)	0.87 (0.12)	0.9	0.42	18.71 (1.72)	0.67 (0.06)	0.89	0.58
S22	0.0	16.6 (3.38)	0.75 (0.3)	0.9	0.38	13.76 (1.67)	0.62 (0.09)	0.93	0.62
S23	0.0	4.22 (5.18)	0.81 (1.06)	0.74	0.13	7.16 (1.86)	0.64 (0.16)	0.94	0.87
S24	0.0	6.95 (5.86)	0.49 (0.91)	0.82	0.23	15.44 (1.82)	0.7 (0.07)	0.92	0.77

RF(3)

Table S6: Decays constants for the transition from the background for the $RF(3)$ segmentation and the $E^1(\sigma = 3) - E^2(\sigma = 4)$ smoothing.

		Transition $BD \to pore$				Transition $BD \to HD$			
$SURF_{ID}$	HD_{off} (% t_{tot})	$\bar{\tau}$ (ps)	a	r^2	N_{trans} (%)	$\bar{\tau}$ (ps)	a	r^2	N_{trans} (%)
S1	0.0	7.14 (2.71)	0.7 (0.33)	0.73	0.2	5.1 (2.24)	0.66 (0.29)	0.94	0.8
S2	0.0	5.76 (5.62)	0.39 (0.72)	0.74	0.19	5.01 (2.29)	0.56 (0.31)	0.94	0.81
S3	0.0	5.87 (5.28)	0.46 (0.81)	0.8	0.25	4.75 (2.07)	0.66 (0.24)	0.93	0.75
S4	0.0	5.31 (4.16)	0.67 (0.83)	0.75	0.2	4.3 (2.49)	0.67 (0.38)	0.94	0.8
S5	0.0	5.71 (2.37)	0.77 (0.19)	0.74	0.2	4.55 (2.04)	0.61 (0.26)	0.93	0.8
S6	0.0	2.83 (3.66)	0.51 (0.8)	0.65	0.15	2.27 (5.45)	0.66 (2.45)	0.95	0.85
S7	0.0	6.13 (2.76)	0.83 (0.17)	0.71	0.17	5.34 (2.11)	0.59 (0.26)	0.94	0.83
S8	0.0	3.32 (16.63)	0.44 (3.28)	0.78	0.2	4.74 (2.27)	0.62 (0.32)	0.94	0.8
S9	0.0	4.87 (11.47)	0.07 (0.2)	0.75	0.18	4.41 (2.43)	0.58 (0.37)	0.94	0.82
S10	0.0	5.68 (2.71)	0.66 (0.4)	0.73	0.19	4.71 (2.0)	0.62 (0.24)	0.94	0.81
S11	0.0	5.09 (2.59)	0.66 (0.39)	0.78	0.23	4.3 (2.15)	0.65 (0.28)	0.94	0.77
S12	0.0	6.08 (2.72)	0.6 (0.39)	0.73	0.21	4.65 (2.23)	0.58 (0.31)	0.94	0.79
S13	0.0	5.8 (2.59)	0.7 (0.33)	0.75	0.2	5.06 (2.59)	0.56 (0.38)	0.94	0.8
S14	0.0	3.01 (6.51)	0.38 (1.15)	0.7	0.15	3.17 (3.1)	0.73 (0.58)	0.95	0.85
S15	0.0	4.63 (3.81)	0.78 (0.62)	0.73	0.16	4.12 (2.51)	0.74 (0.3)	0.95	0.84
S16	0.0	6.59 (2.51)	0.8 (0.17)	0.77	0.22	5.74 (2.0)	0.61 (0.22)	0.93	0.78
S17	0.0	0.97 (3.11)	0.0 (0.0)	0.72	0.14	2.71 (3.99)	0.74 (1.01)	0.95	0.86
S18	0.0	5.5 (3.17)	0.69 (0.52)	0.73	0.18	4.39 (2.31)	0.6 (0.34)	0.94	0.82
S19	0.0	5.67 (2.41)	0.62 (0.32)	0.82	0.27	4.75 (2.17)	0.61 (0.29)	0.94	0.73
S20	0.0	5.7 (5.46)	0.39 (0.7)	0.78	0.19	4.52 (2.24)	0.61 (0.31)	0.95	0.81
S21	0.0	5.05 (4.66)	0.84 (2.19)	0.76	0.2	5.01 (2.3)	0.7 (0.27)	0.94	0.8
S22	0.0	2.12 (13.6)	0.5 (4.32)	0.74	0.16	2.96 (3.64)	0.81 (0.61)	0.95	0.84
S23	0.0	5.34 (3.48)	0.86 (0.25)	0.74	0.17	5.05 (2.14)	0.69 (0.24)	0.94	0.83
S24	0.0	5.56 (3.35)	0.71 (0.55)	0.79	0.19	5.06 (2.29)	0.73 (0.24)	0.95	0.81

THR

Table S7: Decays constants for the transition from the background for the THR segmentation and the $E^1(\sigma = 3) - E^2(\sigma = 4)$ smoothing.

		Transition $BD \to pore$				Transition $BD \to HD$			
$SURF_{ID}$	HD_{off} (% t_{tot})	$\bar{\tau}$ (ps)	a	r^2	N_{trans} (%)	$\bar{\tau}$ (ps)	a	r^2	N_{trans} (%)
S1	0.0	3.41 (4.77)	0.67 (1.28)	0.69	0.13	2.98 (3.01)	0.75 (0.53)	0.96	0.87
S2	0.0	3.5 (12.13)	0.35 (1.96)	0.71	0.13	3.08 (3.82)	0.66 (0.95)	0.96	0.87
S3	0.0	3.01 (23.79)	0.55 (1.84)	0.76	0.15	2.67 (3.79)	0.63 (0.99)	0.96	0.85
S4	0.0	2.46 (18.58)	0.6 (7.34)	0.75	0.14	2.96 (4.03)	0.81 (0.73)	0.96	0.86
S5	0.0	2.57 (6.06)	0.6 (2.19)	0.71	0.12	2.88 (3.33)	0.78 (0.57)	0.96	0.88
S6	0.0	3.23 (3.61)	0.66 (0.85)	0.67	0.13	2.61 (4.02)	0.71 (1.82)	0.96	0.87
S7	0.0	3.54 (2.69)	0.77 (0.35)	0.63	0.1	3.08 (3.39)	0.6 (0.77)	0.97	0.9
S8	0.0	1.04 (0.27)	0.03 (0.0)	0.74	0.13	3.16 (3.12)	0.68 (0.66)	0.96	0.87
S9	0.0	1.08 (0.28)	0.04 (0.0)	0.76	0.14	3.12 (3.46)	0.58 (0.78)	0.96	0.86
S10	0.0	1.81 (21.73)	0.33 (4.11)	0.72	0.12	3.1 (2.95)	0.66 (0.61)	0.96	0.88
S11	0.0	3.0 (11.86)	0.5 (3.24)	0.79	0.15	2.74 (3.79)	0.65 (0.99)	0.96	0.85
S12	0.0	3.52 (6.67)	0.92 (5.19)	0.74	0.13	3.1 (3.17)	0.72 (0.65)	0.96	0.87
S13	0.0	3.69 (4.09)	0.87 (0.42)	0.73	0.14	3.14 (3.45)	0.67 (0.79)	0.96	0.86
S14	0.0	3.13 (11.05)	0.15 (0.64)	0.72	0.13	3.04 (3.52)	0.79 (0.6)	0.96	0.87
S15	0.0	3.96 (4.38)	0.88 (0.46)	0.7	0.12	3.05 (3.68)	0.76 (1.72)	0.96	0.88
S16	0.0	3.53 (2.96)	0.79 (0.36)	0.73	0.14	3.26 (2.91)	0.75 (0.47)	0.96	0.86
S17	0.0	3.32 (1.81)	0.53 (0.32)	0.76	0.12	3.05 (3.54)	0.71 (0.8)	0.96	0.88
S18	0.0	3.64 (4.09)	0.86 (0.48)	0.73	0.12	2.13 (6.73)	0.51 (2.1)	0.97	0.88
S19	0.0	3.06 (5.33)	0.88 (0.76)	0.81	0.18	2.94 (3.05)	0.71 (0.62)	0.96	0.82
S20	0.0	3.44 (11.85)	0.96 (0.61)	0.77	0.13	3.01 (2.9)	0.75 (0.48)	0.96	0.87
S21	0.0	0.98 (0.25)	0.01 (0.0)	0.74	0.14	3.16 (3.41)	0.74 (0.7)	0.96	0.86
S22	0.0	2.2 (63.8)	0.43 (16.06)	0.76	0.15	3.37 (3.37)	0.79 (0.55)	0.96	0.85
S23	0.0	0.99 (0.23)	0.01 (0.0)	0.73	0.12	3.52 (3.25)	0.8 (0.43)	0.96	0.88
S24	0.0	1.0 (0.26)	0.02 (0.0)	0.78	0.15	3.24 (3.16)	0.81 (0.4)	0.96	0.85

Sequential reclassification of HD regions into BD

Table S8: Full iterations for the re-attribution of the HD regions to the background class for a reference surface (S10) using the $RF(5)$-$E^1(\sigma = 4) - E^2(\sigma = 4)$ segmentation.

	Transition $BD \rightarrow pore$					Transition $BD \rightarrow HD$				
HD_{off}	$\tau_1(ps)$	$\tau_2(ps)$	a	r^2	$N_{trans}(\%)$	$\tau_1(ps)$	$\tau_2(ps)$	a	r^2	$N_{trans}(\%)$
0.0	16.42 (2.38)	4.11 (5.36)	0.846 (0.195)	0.88	0.29	14.73 (1.94)	2.07 (0.99)	0.637 (0.097)	0.94	0.71
0.1	16.73 (2.26)	3.99 (5.38)	0.857 (0.178)	0.88	0.29	15.2 (1.99)	2.13 (0.97)	0.626 (0.095)	0.93	0.71
0.2	18.24 (2.61)	4.67 (5.36)	0.832 (0.193)	0.88	0.3	16.22 (2.15)	2.3 (0.96)	0.604 (0.093)	0.93	0.7
0.3	18.51 (2.09)	3.9 (5.13)	0.869 (0.14)	0.88	0.31	17.13 (2.06)	2.19 (0.9)	0.614 (0.082)	0.93	0.69
0.4	18.73 (2.05)	3.83 (5.05)	0.872 (0.133)	0.88	0.32	17.5 (2.09)	2.23 (0.9)	0.611 (0.081)	0.93	0.68
0.5	18.93 (1.94)	3.63 (4.95)	0.88 (0.121)	0.89	0.32	18.14 (2.07)	2.21 (0.88)	0.614 (0.076)	0.93	0.68
0.6	19.42 (2.13)	4.14 (5.99)	0.883 (0.139)	0.89	0.33	18.79 (1.98)	2.13 (0.87)	0.632 (0.071)	0.93	0.67
0.7	20.16 (2.42)	4.79 (6.6)	0.873 (0.161)	0.89	0.34	19.86 (2.08)	2.25 (0.86)	0.617 (0.069)	0.92	0.66
0.8	22.77 (3.75)	6.95 (6.7)	0.795 (0.242)	0.89	0.35	20.55 (2.14)	2.31 (0.85)	0.609 (0.067)	0.92	0.65
0.9	22.75 (3.87)	7.11 (7.0)	0.794 (0.255)	0.89	0.35	20.47 (2.11)	2.29 (0.86)	0.616 (0.067)	0.92	0.65
1.0	23.56 (3.55)	6.9 (6.82)	0.81 (0.218)	0.89	0.36	21.26 (2.08)	2.3 (0.88)	0.627 (0.064)	0.92	0.64
1.1	24.13 (3.54)	7.01 (6.87)	0.812 (0.212)	0.9	0.37	21.63 (2.07)	2.29 (0.87)	0.63 (0.062)	0.92	0.63
1.2	26.1 (4.22)	8.26 (7.65)	0.793 (0.244)	0.91	0.39	22.69 (1.96)	2.14 (0.85)	0.652 (0.056)	0.92	0.61
1.3	26.23 (3.44)	7.29 (7.23)	0.828 (0.187)	0.91	0.39	23.17 (1.93)	2.08 (0.84)	0.66 (0.054)	0.92	0.61
1.4	26.32 (3.42)	7.33 (7.58)	0.835 (0.187)	0.91	0.4	23.55 (1.89)	2.05 (0.86)	0.671 (0.052)	0.92	0.6
1.6	26.83 (3.48)	7.48 (7.5)	0.832 (0.185)	0.91	0.4	23.8 (1.89)	2.06 (0.87)	0.675 (0.052)	0.92	0.6
1.7	26.94 (3.54)	7.63 (7.83)	0.834 (0.191)	0.91	0.4	24.11 (1.85)	2.01 (0.87)	0.683 (0.05)	0.92	0.6
1.8	27.09 (3.34)	7.38 (8.07)	0.849 (0.177)	0.92	0.41	24.99 (1.82)	1.96 (0.86)	0.694 (0.048)	0.92	0.59
2.0	29.82 (5.08)	10.09 (8.57)	0.774 (0.266)	0.92	0.42	26.07 (1.91)	2.12 (0.88)	0.682 (0.048)	0.92	0.58
2.1	30.38 (4.56)	9.76 (9.16)	0.808 (0.234)	0.92	0.44	27.27 (1.89)	2.12 (0.9)	0.696 (0.045)	0.91	0.56
2.2	33.81 (6.52)	12.37 (8.85)	0.724 (0.303)	0.92	0.45	28.26 (2.01)	2.34 (0.92)	0.681 (0.046)	0.91	0.55
2.3	34.14 (6.6)	12.64 (9.45)	0.733 (0.311)	0.93	0.46	28.77 (1.99)	2.27 (0.91)	0.685 (0.045)	0.91	0.54
2.6	36.35 (7.91)	14.12 (9.28)	0.686 (0.344)	0.92	0.47	29.25 (2.01)	2.3 (0.9)	0.683 (0.044)	0.91	0.53
2.8	37.68 (6.76)	13.57 (8.85)	0.719 (0.276)	0.92	0.49	29.62 (2.02)	2.31 (0.9)	0.682 (0.044)	0.91	0.51
3.0	38.92 (6.7)	13.84 (8.88)	0.723 (0.263)	0.92	0.5	30.36 (2.06)	2.38 (0.9)	0.676 (0.043)	0.91	0.5
3.1	39.45 (6.6)	13.87 (8.78)	0.725 (0.253)	0.93	0.5	31.01 (2.07)	2.38 (0.9)	0.676 (0.042)	0.91	0.5
3.2	39.38 (5.34)	12.53 (8.72)	0.775 (0.201)	0.93	0.51	31.99 (2.13)	2.5 (0.91)	0.671 (0.042)	0.9	0.49
3.4	40.07 (4.77)	11.87 (8.49)	0.797 (0.171)	0.93	0.52	32.55 (2.13)	2.46 (0.89)	0.672 (0.041)	0.9	0.48
3.5	41.75 (4.73)	12.06 (8.21)	0.795 (0.159)	0.93	0.54	32.96 (2.15)	2.5 (0.89)	0.669 (0.041)	0.89	0.46
4.3	41.55 (4.63)	11.94 (8.58)	0.807 (0.158)	0.93	0.54	33.86 (2.16)	2.51 (0.89)	0.671 (0.04)	0.89	0.46
4.4	42.84 (4.61)	12.14 (8.57)	0.808 (0.151)	0.93	0.55	34.52 (2.2)	2.61 (0.91)	0.669 (0.04)	0.89	0.45
4.7	44.22 (4.35)	11.85 (8.26)	0.817 (0.134)	0.93	0.56	35.41 (2.24)	2.7 (0.92)	0.666 (0.039)	0.88	0.44
5.1	44.34 (4.51)	12.19 (8.42)	0.812 (0.141)	0.93	0.56	35.15 (2.21)	2.71 (0.95)	0.675 (0.04)	0.89	0.44
5.3	46.17 (4.46)	12.39 (8.36)	0.815 (0.132)	0.94	0.58	35.99 (2.25)	2.78 (0.95)	0.671 (0.039)	0.88	0.42
5.5	47.8 (5.29)	14.13 (8.64)	0.783 (0.156)	0.94	0.58	38.06 (2.37)	3.05 (0.99)	0.664 (0.039)	0.88	0.42
6.6	48.71 (4.87)	13.56 (8.43)	0.799 (0.137)	0.94	0.59	39.34 (2.4)	3.11 (0.99)	0.663 (0.038)	0.87	0.41
6.7	51.41 (5.04)	14.37 (8.66)	0.797 (0.135)	0.94	0.61	41.06 (2.47)	3.17 (0.97)	0.653 (0.037)	0.86	0.39
7.0	52.32 (4.69)	13.78 (8.31)	0.808 (0.119)	0.94	0.62	42.77 (2.54)	3.3 (0.97)	0.648 (0.036)	0.86	0.38
7.4	52.31 (4.55)	13.52 (8.45)	0.817 (0.116)	0.94	0.62	43.45 (2.52)	3.31 (0.99)	0.655 (0.035)	0.86	0.38
8.1	54.36 (4.44)	13.62 (8.57)	0.825 (0.107)	0.94	0.64	45.05 (2.63)	3.51 (0.99)	0.643 (0.035)	0.85	0.36
8.3	55.19 (4.21)	13.17 (8.45)	0.835 (0.098)	0.95	0.65	46.64 (2.73)	3.71 (1.0)	0.636 (0.035)	0.85	0.35
9.1	55.31 (3.86)	12.34 (8.75)	0.856 (0.088)	0.95	0.66	48.6 (2.78)	4.06 (1.11)	0.648 (0.035)	0.85	0.34
9.5	56.08 (3.95)	12.7 (8.88)	0.854 (0.09)	0.95	0.66	49.49 (2.68)	3.86 (1.1)	0.663 (0.034)	0.85	0.34
9.8	57.78 (4.01)	13.1 (9.09)	0.855 (0.088)	0.95	0.67	49.96 (2.66)	3.82 (1.1)	0.668 (0.033)	0.85	0.33
11.4	59.84 (4.27)	14.07 (9.16)	0.845 (0.092)	0.95	0.68	51.38 (2.72)	4.02 (1.13)	0.665 (0.033)	0.85	0.32
13.7	65.78 (7.16)	20.82 (9.81)	0.744 (0.154)	0.94	0.68	51.26 (2.73)	4.03 (1.13)	0.665 (0.033)	0.85	0.32
14.0	76.13 (12.35)	28.59 (10.06)	0.609 (0.219)	0.95	0.7	49.52 (2.5)	3.79 (1.24)	0.707 (0.033)	0.85	0.3
14.4	75.95 (12.38)	29.51 (11.96)	0.643 (0.241)	0.95	0.73	51.56 (2.34)	3.37 (1.25)	0.738 (0.03)	0.83	0.27
14.5	77.47 (14.19)	32.39 (15.04)	0.654 (0.298)	0.96	0.77	56.43 (2.34)	3.62 (1.46)	0.769 (0.029)	0.8	0.23
15.2	87.03 (23.87)	39.37 (14.13)	0.503 (0.374)	0.96	0.78	56.7 (2.29)	3.61 (1.58)	0.787 (0.028)	0.8	0.22
16.0	88.36 (21.61)	39.28 (14.68)	0.54 (0.351)	0.96	0.8	59.31 (2.43)	3.99 (1.54)	0.768 (0.028)	0.79	0.2
19.6	101.72 (28.08)	45.0 (14.21)	0.472 (0.341)	0.96	0.85	58.9 (2.27)	3.35 (1.47)	0.787 (0.026)	0.74	0.15
23.9	106.57 (26.42)	45.53 (13.84)	0.488 (0.304)	0.96	0.87	63.62 (2.27)	3.2 (1.49)	0.801 (0.024)	0.72	0.13
27.8	112.65 (26.37)	46.37 (12.98)	0.481 (0.27)	0.96	0.88	64.29 (2.19)	2.84 (1.46)	0.813 (0.023)	0.72	0.12
28.0	117.77 (25.36)	47.2 (13.1)	0.5 (0.25)	0.96	0.9	66.6 (2.41)	3.3 (1.3)	0.767 (0.024)	0.64	0.1
36.3	118.59 (27.71)	48.85 (14.02)	0.491 (0.274)	0.96	0.9	72.58 (2.39)	3.19 (1.4)	0.794 (0.022)	0.65	0.1
36.5	118.34 (27.65)	49.2 (14.65)	0.501 (0.282)	0.96	0.9	77.71 (2.3)	3.23 (1.85)	0.844 (0.021)	0.67	0.1
42.7	130.05 (25.72)	49.64 (13.1)	0.511 (0.22)	0.96	0.92	86.17 (2.83)	5.32 (1.87)	0.784 (0.023)	0.55	0.08
52.9	158.42 (36.99)	55.07 (10.81)	0.407 (0.183)	0.96	0.94	88.48 (2.62)	3.42 (1.38)	0.787 (0.019)	0.49	0.06
58.9	173.22 (40.51)	56.78 (10.22)	0.391 (0.164)	0.96	0.94	107.91 (4.06)	11.37 (2.78)	0.74 (0.028)	0.47	0.06

References

(1) Du, J.; Cormack, A. The medium range structure of sodium silicate glasses: a molecular dynamics simulation. **2004**, *349*, 66–79.

(2) Le, T.; Striolo, A.; Cole, D. R. CO2–C4H10 mixtures simulated in silica slit pores: relation between structure and dynamics. *The Journal of Physical Chemistry C* **2015**, *119*, 15274–15284.

(3) Turchi, M.; Galmarini, S.; Lunati, I. Learning Adsorption Patterns on Amorphous Surfaces. *Journal of Chemical Theory and Computation* **2024**, *20*, 7597–7610.

(4) Lane, J. M. D. Cooling rate and stress relaxation in silica melts and glasses via microsecond molecular dynamics. *Phys. Rev. E* **2015**, *92*, 012320.

(5) Wimalasiri, P. N.; Nguyen, N. P.; Senanayake, H. S.; Laird, B. B.; Thompson, W. H. Amorphous Silica Slab Models with Variable Surface Roughness and Silanol Density for Use in Simulations of Dynamics and Catalysis. *The Journal of Physical Chemistry C* **2021**, *125*, 23418–23434.

(6) Ramsheh, S. M.; Turchi, M.; Perera, S.; Schade, A.; Okhrimenko, D.; Stipp, S.; Solvang, M.; Walsh, T.; Andersson, M. Prediction of the surface chemistry of calcium aluminosilicate glasses. *Journal of Non-Crystalline Solids* **2023**, *620*, 122597.

(7) Turchi, M.; Perera, S.; Ramsheh, S.; Popel, A.; Okhrimenko, D.; Stipp, S.; Solvang, M.; Andersson, M.; Walsh, T. Predicted structures of calcium aluminosilicate glass as a model for stone wool fiber: effects of composition and interatomic potential. *Journal of Non-Crystalline Solids* **2021**, *567*, 120924.

(8) Du, J.; Cormack, A. N. Molecular Dynamics Simulation of the Structure and Hydroxylation of Silica Glass Surfaces. *Journal of the American Ceramic Society* **2005**, *88*, 2532–2539, Publisher: John Wiley & Sons, Ltd.

(9) Turchi, M.; Galmarini, S.; Lunati, I. Amorphous matters: Heterogeneity and defects of nanopore silica surfaces enhance CO2 adsorption. *Journal of Non-Crystalline Solids* **2024**, *624*, 122709.

(10) Vuković, F.; Garcia, N.; Perera, S.; Turchi, M.; Andersson, M.; Solvang, M.; Raiteri, P.; Walsh, T. Atomistic simulations of calcium aluminosilicate interfaced with liquid water. *The Journal of Chemical Physics* **2023**, *159*.

(11) Guttman, L. Ring structure of the crystalline and amorphous forms of silicon dioxide. *Journal of Non-Crystalline Solids;(Netherlands)* **1990**, *116*.

(12) Cygan, R. T.; Liang, J.-J.; Kalinichev, A. G. Molecular Models of Hydroxide, Oxyhydroxide, and Clay Phases and the Development of a General Force Field. *The Journal of Physical Chemistry B* **2004**, *108*, 1255–1266, Publisher: American Chemical Society.

(13) Zhuravlev, L. The surface chemistry of amorphous silica. Zhuravlev model. *Colloids and Surfaces A: Physicochemical and Engineering Aspects* **2000**, *173*, 1–38.

(14) Fogarty, J. C.; Aktulga, H. M.; Grama, A. Y.; Van Duin, A. C.; Pandit, S. A. A reactive molecular dynamics simulation of the silica-water interface. *The Journal of chemical physics* **2010**, *132*.

(15) Inaki, Y.; Yoshida, H.; Yoshida, T.; Hattori, T. Active sites on mesoporous and amorphous silica materials and their photocatalytic activity: an investigation by FTIR, ESR, VUV- UV and photoluminescence spectroscopies. *The Journal of Physical Chemistry B* **2002**, *106*, 9098–9106.

(16) Hamid, M.; Firmansyah, M.; Triwahyono, S.; Jalil, A. A.; Mukti, R.; Febriyanti, E.; Suendo, V.; Setiabudi, H.; Mohamed, M.; Nabgan, W. Oxygen vacancy-rich mesoporous silica KCC-1 for CO2 methanation. *Applied Catalysis A: General* **2017**, *532*, 86–94.

(17) Cygan, R. T.; Romanov, V. N.; Myshakin, E. M. Molecular simulation of carbon dioxide capture by montmorillonite using an accurate and flexible force field. *The Journal of Physical Chemistry C* **2012**, *116*, 13079–13091.

(18) Harris, J. G.; Yung, K. H. Carbon Dioxide's Liquid-Vapor Coexistence Curve And Critical Properties as Predicted by a Simple Molecular Model. *The Journal of Physical Chemistry* **1995**, *99*, 12021–12024, Publisher: American Chemical Society.

(19) Crabtree, J. C.; Molinari, M.; Parker, S. C.; Purton, J. A. Simulation of the Adsorption and Transport of CO2 on Faujasite Surfaces. *The Journal of Physical Chemistry C* **2013**, *117*, 21778–21787, Publisher: American Chemical Society.

(20) Purton, J.; Crabtree, J. C.; Parker, S. DL_MONTE: a general purpose program for parallel Monte Carlo simulation. *Molecular Simulation* **2013**, *39*, 1240–1252.

(21) Bourg, I. C.; Steefel, C. I. Molecular Dynamics Simulations of Water Structure and Diffusion in Silica Nanopores. *The Journal of Physical Chemistry C* **2012**, *116*, 11556–11564, Publisher: American Chemical Society.

(22) Ohno, H.; Kohara, S.; Umesaki, N.; Suzuya, K. High-energy X-ray diffraction studies of non-crystalline materials. *Journal of non-crystalline solids* **2001**, *293*, 125–135.

(23) Hudon, P.; Jung, I.-H.; Baker, D. R. Melting of β-quartz up to 2.0 GPa and thermodynamic optimization of the silica liquidus up to 6.0 GPa. *Physics of the Earth and Planetary Interiors* **2002**, *130*, 159–174.

(24) Leroch, S.; Wendland, M. Simulation of forces between humid amorphous silica surfaces: A comparison of empirical atomistic force fields. *The Journal of Physical Chemistry C* **2012**, *116*, 26247–26261.

(25) Takei, T.; Yamazaki, A.; Watanabe, T.; Chikazawa, M. Water adsorption properties

on porous silica glass surface modified by trimethylsilyl groups. *Journal of colloid and interface science* **1997**, *188*, 409–414.

(26) Rimsza, J.; Jones, R.; Criscenti, L. Surface structure and stability of partially hydroxylated silica surfaces. *Langmuir* **2017**, *33*, 3882–3891.

(27) Nosé, S. A molecular dynamics method for simulations in the canonical ensemble. *Molecular Physics* **1984**, *52*, 255–268.

(28) Hoover, W. G. Canonical dynamics: Equilibrium phase-space distributions. *Phys. Rev. A* **1985**, *31*, 1695–1697.

(29) Hockney, R. W.; Eastwood, J. W. *Computer simulation using particles*; crc Press, 2021.

(30) Cerutti, D. S.; Duke, R. E.; Darden, T. A.; Lybrand, T. P. Staggered Mesh Ewald: An Extension of the Smooth Particle-Mesh Ewald Method Adding Great Versatility. *Journal of Chemical Theory and Computation* **2009**, *5*, 2322–2338.

(31) Neelov, A.; Holm, C. Interlaced P3M algorithm with analytical and ik-differentiation. *The Journal of Chemical Physics* **2010**, *132*, 234103.
